# Hafting of Middle Paleolithic tools in Latium (central Italy): New data from Fossellone and Sant’Agostino caves

**DOI:** 10.1371/journal.pone.0213473

**Published:** 2019-06-20

**Authors:** Ilaria Degano, Sylvain Soriano, Paola Villa, Luca Pollarolo, Jeannette J. Lucejko, Zenobia Jacobs, Katerina Douka, Silvana Vitagliano, Carlo Tozzi

**Affiliations:** 1 Department of Chemistry and Industrial Chemistry, Università di Pisa, Pisa, Italy; 2 ArScAn, AnTET, CNRS, Maison de l’Archéologie et de l’Ethnologie, Université Paris Nanterre, Nanterre Cedex, France; 3 University of Colorado Museum, Boulder, Colorado, United States of America; 4 Istituto Italiano di Paleontologia Umana, Rome, Italy; 5 School of Geography, Archaeology and Environmental Studies, University of the Witwatersrand, Johannesburg, South Africa; 6 Laboratoire Archéologie et Peuplement de l’Afrique, University of Geneva, Genève, Switzerland; 7 Centre for Archaeological Science, School of Earth, Atmospheric and Life Sciences, University of Wollongong, Wollongong, Australia; 8 Australian Research Council (ARC) Centre of Excellence for Australian Biodiversity and Heritage, University of Wollongong, Wollongong, Australia; 9 Department of Archaeology, Max Planck Institute for the Science of Human History, Jena, Germany; 10 Oxford Radiocarbon Accelerator Unit, Research Laboratory for Archaeology and the History of Art, University of Oxford, Oxford, United Kingdom; 11 Istituto Italiano di Paleontologia Umana, Museo Civico di Zoologia, Rome, Italy; 12 Dipartimento di Civiltà e Forme del Sapere, Università di Pisa, Pisa, Italy; Universita degli Studi di Milano, ITALY

## Abstract

Hafting of stone tools was an important advance in the technology of the Paleolithic. Evidence of hafting in the Middle Paleolithic is growing and is not limited to points hafted on spears for thrusting or throwing. This article describes the identification of adhesive used for hafting on a variety of stone tools from two Middle Paleolithic caves in Latium, Fossellone Cave and Sant’Agostino Cave. Analysis of the organic residue by gas chromatography/mass spectrometry shows that a conifer resin adhesive was used, in one case mixed with beeswax. Contrary to previous suggestions that the small Middle Paleolithic tools of Latium could be used by hand and that hafting was not needed since it did not improve their functionality, our evidence shows that hafting was used by Neandertals in central Italy. Ethnographic evidence indicates that resin, which dries when exposed to air, is generally warmed by exposure to a small fire thus softened to be molded and pushed in position in the haft. The use of resin at both sites suggests regular fire use, as confirmed by moderate frequencies of burnt lithics in both assemblages. Lithic analysis shows that hafting was applied to a variety of artifacts, irrespective of type, size and technology. Prior to our study evidence of hafting in the Middle Paleolithic of Italy was limited to one case only.

## Introduction: The identification of hafting

The hafting of stone tools was an important advance in the technological evolution of Paleolithic humans. Joining a handle to a knife or scraper and attaching a sharp point to a wooden shaft made stone tools more efficient and easier to use [1, 2]. A comprehensive review of the various methods for the identification of hafting in prehistory can be found in [1]. For the purpose of this paper we present a brief review of categories of evidence.

The best and direct type of evidence for hafting is the finding of intact hafted tools. However, these are rare finds from sites with exceptional preservation, and all are dated to the Holocene or the end of the Pleistocene. One example is from Yukon, Canada. The melting ice patches in southwest Yukon have yielded many wooden arrows, bone and antler points [3]. One of these projectile weapons was an antler point slotted for microblade inserts held in place by an adhesive identified as spruce resin by gas chromatography/mass spectrometry (GC/MS) [4].

Similar examples of wooden arrows, antler and bone points with the flint elements still in place are dated to the Mesolithic [5] and two at least are dated to the late Upper Paleolithic [6,7]. GC/MS was used to identify the adhesive on weapons of Ötzi, the Alpine Iceman that died about 5200 BP at the border between Austria and Italy [1, 8].

Older artifacts have until now provided only indirect evidence of hafting. With the exception of bone tubes from Buran-Kaya III level C dated to the transition Middle to Upper Paleolithic in Crimea and most likely used as handles [9], the haft of Middle Paleolithic tools was likely made of wood and its usual decay in prehistoric sites means that only indirect lines of evidence are available to archaeologists. One of the earlier proposed lines of evidence was the presence of a peculiar patina pattern observed on a Mousterian side scraper with a whitish patina on most of the tool faces but with the original stone color on the proximal and a lateral edge [10]. Experimental work proved that a patina can result from exposure to atmosphere in a relatively short time while the hafted (hence protected) area retains the original dark stone color, thus indicating the location of the haft. A similar observation was done on a Still Bay point from Blombos which was axially hafted [11].

Other hafting traces depend on the way of arranging a tool in the haft. Thus microwear analysts look at microscopic scars, striations and polishes on the artifact edge where a distinctive pattern may be left by hafts and binding due to friction between the haft components [1, 12–16].

Another category of evidence comes from the identification of impact scars on spear tips and projectile points [11, 16,17,18] and references therein] and on laterally hafted microliths [19]. Morphological features such as tangs, bilateral notches, shoulders, base thinning, fluting on Clovis points, thin tips and flat profile of triangular flakes, or tip cross-sectional areas are features often used in combination with other lines of evidence to identify hafted points [20–22]. Clearly the presence of adhesive materials on the side or base of an artifact strengthens the reliability of inferences based on microwear, impact scars or morphological features.

### Resin and tar residues

If the adhesive, preserved on a tool as a black residue, is an organic material, its molecular composition can be identified by GC/MS. The advantage of GC/MS over microscopic examinations is that it is possible to conclusively identify the source of resinous material and identify compounds present in a mixture [23]. Microscopic examination, if not substantiated by chemical analysis cannot be considered conclusive for the identification of resinous material [24, 25]. GC/MS is currently considered the most robust method for the taxonomic characterization of organic adhesives [26]. This procedure has been successfully used in South African Middle Stone Age and Later Stone Age sites such as Sibudu, Diepkloof and Border Cave [27–31].

Middle Paleolithic examples of hafting technology, based on data provided by GC/MS, are several. The earliest evidence comes from Campitello Quarry in central Italy where a pitch made from birch bark was found on the proximal part of two flakes found in association with the bones of a young elephant (*Palaeoloxodon antiquus*) and several micromammals [32]. The micromammals date the site to a time shortly preceding the end of the Middle Pleistocene, a cool stadial episode before isotope stage 6. This implies most likely MIS 7.2, a cool but not very cold period within MIS 7, dated to 206–201 ka [32–34, 1; *contra* 26].

Two lumps of birch bark pitch, verified by combined GC/MS, come from the site of Königsaue in Germany, which has an estimated age of 80,000. One of the pieces has a negative imprint of a wooden haft [35–36] (see also [37]). Heat-treated natural bitumen, identified by GC/MS, was used at the site of Umm-el-Tlel (Syria) for hafting purposes in layers dated 42,000 and 70,000 years ago [38, 39]. Evidence of bitumen on a Mousterian point and two Levallois flakes from the site of Hummal (Syria) was also found by GC/MS confirming a previous microscopic analysis, based on SEM-EDS, FTIR and Raman microscopy [40–41]. Bitumen was also identified by GC/MS as hafting residue on a retouched flake from a late Mousterian layer at a cave in Romania [42].

All the described different methods present some problems. We briefly discuss here the two most commonly used methods and the reliability of their results.

### Problems in the identification of hafting by microscopic analysis

The advantage of this method, when low-power and high-power microscopy are used, is that in assemblages with a low degree of postdepositional modification a large number of tools can be analyzed. Combining traces of hafting with evidence of use-wear traces on the working edges can provide plausible results. This is the case of Veerle Rots’ analysis of stone tools from the Middle Paleolithic site of Biache [16]. Out of 105 tools that showed possible evidence of use, use-wear traces were identified with reasonable certainty for 65 tool edges; the prehensile mode was inferred. However the morphology of some residues is difficult to interpret [43]. Microscopic analysis is a subjective technique [44] since observations of wear traces vary between researchers. Blind tests have been used as an independent verification. Blind test applied to lithic microwear analyses have shown that the analyst’s experience is an important factor but that a certain level of uncertainty and inaccuracies cannot be eliminated [44, 45]. The importance of considering the depositional context of examined tools to avoid misidentification due to incidental deposition of various residues on the tool edge is also stressed [43, 46, 47].

### Problems with the gas chromatography/mass spectrometry method

The main problem of this method, considered the most reliable method for the identification of organic adhesives [26] comes from the low level of preservation of organic residues. GC/MS analysis of one quartz flake in the Late Howiesons Poort layers of Diepkloof attested to the exploitation of resin from *Podocarpus elongatus* used for lateral hafting [30]. Twenty-eight other pieces have similar black residue in a lateral position, opposite to the sharpened edge. These were only macroscopically analyzed thus the hypothesis that those pieces were hafted can be considered as a proposition with limited certainty. Visual examination of 15,000 lithic artifacts from HP layers did not reveal any other evidence of artifacts with hafting adhesive [30].

Likewise, of six artifacts from MSA assemblages of Sibudu and Rose Cottage selected by a lithic specialist from hundreds of artifacts because they had ochre lines along a backed edge or had been described with residues, only two gave significant results [27]. More than 1000 artifacts come from layers of complex VI 3 at Umm el Tlel; of these 11 have black residue identified as bitumen by GC/MS. About 30 flakes also had traces of black residue but the limited amount was insufficient for chemical analysis [39]. Thus while the chemical method is reliable and precise, the low level of organic residue preservation may restrict the interpretation of hafting to a small set of tools. In our study we used GC/MS which has greater analytical sensitivity than FTIR (Fourier Transform Infrared Spectroscopy [23] in quantitative measurements. The method is destructive but it requires only minute amount of residue (see “Sampling for GC/MS analysis”).

### Objectives

**Fig 1:**
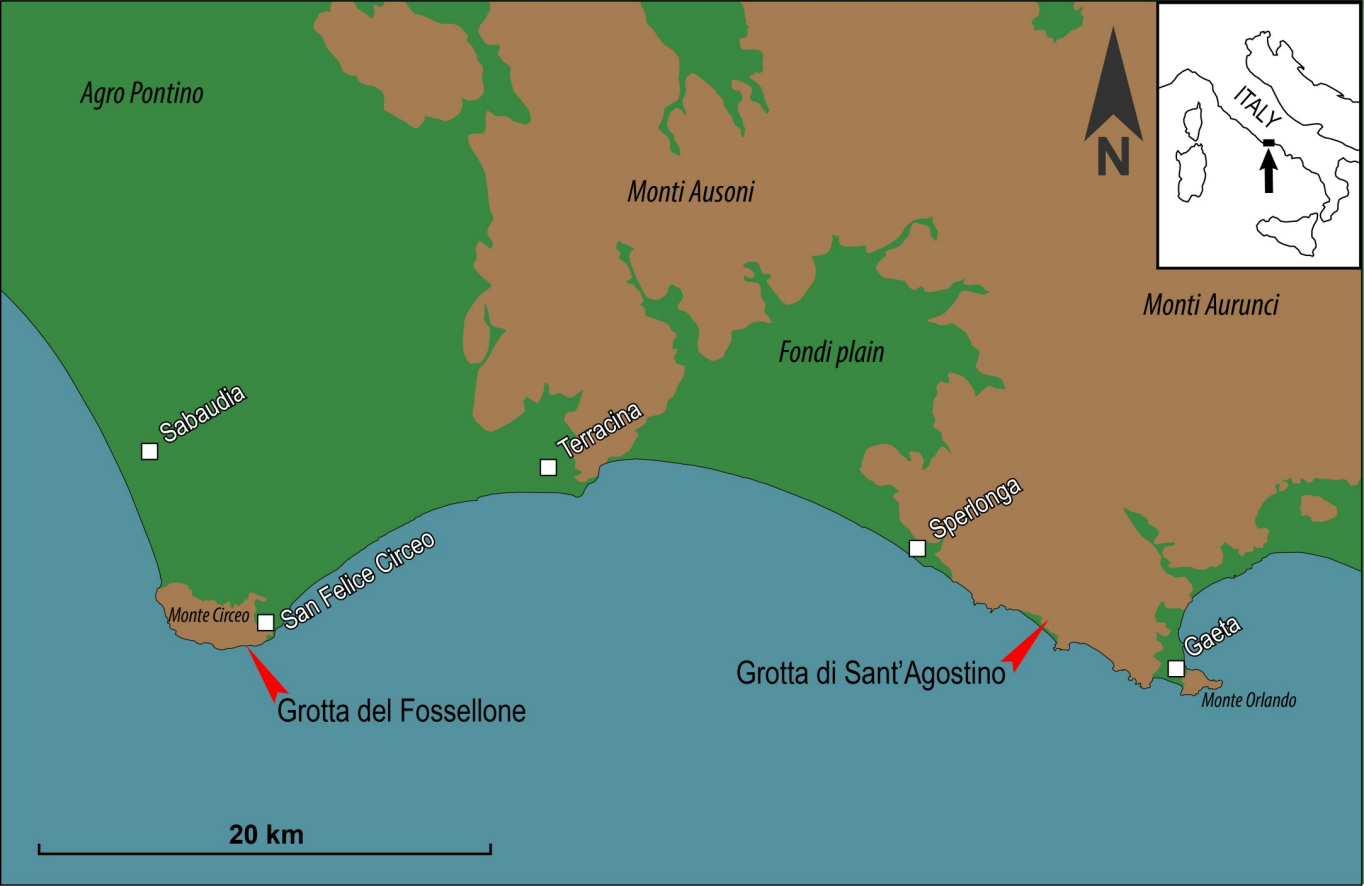
Map of Grotta del Fossellone (41°13’26.29” N; 13°04’50.53” E) and Grotta di Sant’Agostino (41°14’01” N; 13°30’13.08” E).

The objectives of our paper are: (a) to present the results of GC/MS analyses of organic residue on a number of stone artifacts from two Middle Paleolithic cave sites in central Italy, namely Grotta del Fossellone (layer 23 alpha) and Grotta di Sant’Agostino (layer A1), (Fig 1); (b) to describe the lithic industry, the context and dating of the two sites; this background information is needed to support the age and technology of the artifacts, hence it precedes the residue analysis; (c) to show that the organic residue is not the result of incidental deposition; (d) to examine previous suggestions that the small tools of Latium were used by hand and that hafting was not needed [48].

## Materials and methods

### Permits and repositories

The Fossellone collections are housed in the Pigorini National Museum of Prehistory and Ethnography in Rome. Permits to study, to take photos of the materials and to get faunal samples for dating were obtained by the Soprintendenza of the Pigorini Museum (Prot. MBAC-S-MNPE Pigorini CL 25-02-03/1.5 and CL 25-02-04/5 and MBAC-S-MNPE Pigorini 0002338 24/06//2010 CL 25.02.03/1.5). Permit to clean the upper part of the Fossellone stratigraphic section with Middle Paleolithic and Aurignacian deposits and to take sediment samples for OSL dating was obtained by the Soprintendenza per i Beni Archeologici del Lazio (Prot. DG 6583, Class.34-31-02/414.1). Faunal materials for dating were kindly provided by Antonio Tagliacozzo of the Pigorini Museum.

The Sant’Agostino collections, except for a small number of pieces at the Museo Pigorini in Rome, are housed in the storage area of the Dipartimento di Civiltà e Forme del Sapere, University of Pisa, via Trieste 40, under the care of Prof. Carlo Tozzi, a co-author of this study. Hence a permit was not required.

### Specimen numbers

The Fossellone lithics from layer 23 alpha had the layer indicated but no catalogue number. We assigned individual number in sequential order to retouched pieces, flakes and cores individually bagged in reusable zipper bags (Minigrips) with preprinted labels.

All the Sant’Agostino pieces from layer A1 had catalogue numbers assigned by Carlo Tozzi for his study published in 1970.

### Chemical analysis

The analytical procedures and apparatus are in S2 File.

### Grotta del Fossellone

#### Context and stratigraphy

**Fig 2:**
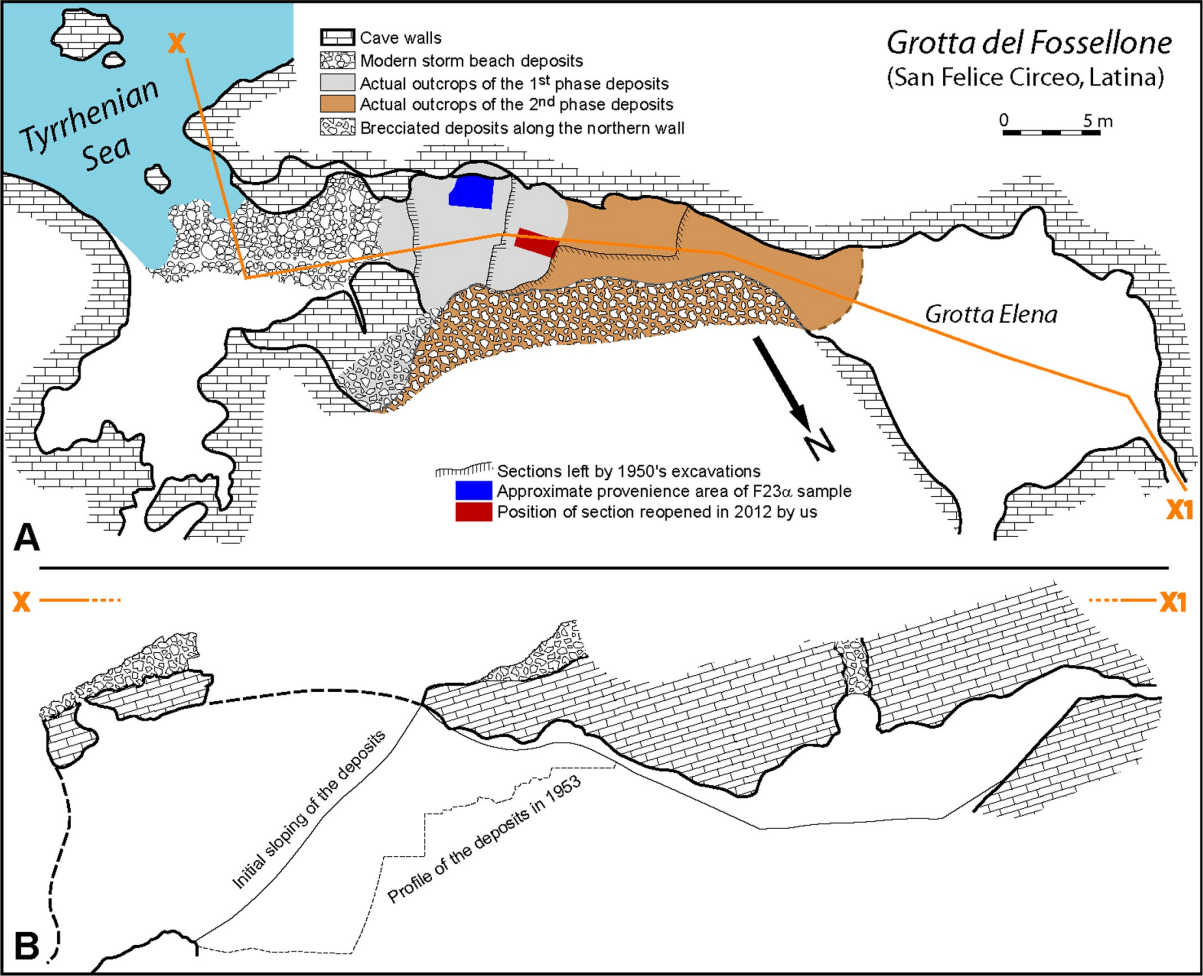
Plan and section of Grotta del Fossellone. **(**A) Plan of the cave with location of the 2012 fieldwork, intact remnants of Pleistocene deposits and approximate provenience of F23α sample from the 1947–1953 excavation. Modified from [50]. (B) Schematic longitudinal section of the cave showing the large opening in the roof, modified from [49]. Courtesy of the Istituto Italiano di Paleontologia Umana.

Grotta del Fossellone opens onto the Mediterranean Sea at the foot of Monte Circeo. It owes its name (literally Great Ditch cave) to the wide circular opening that affects the limestone roof. It was excavated by the Institute of Human Paleontology in Rome under the supervision of A.C. Blanc [49], (Fig 2). When the cave was first explored in 1936, its only access was on a wide steep slope, covered with vegetation, descending to the sea. Subsequently, as the excavations progressed, an upper access was established along a gap between the infilling of the cave and the north wall. This gap ends in the deep part of the cavity called Grotta Elena. In its proximal part, the north wall is divided into small cavities and secondary tunnels, which were test excavated in 1937 and 1938. Systematic excavation did not really begin until after World War II, in 1947.

The Pleistocene infilling of the cave was originally up to the level of the roof opening. It can be divided into two phases. During the first phase, various layers accumulated over almost 6.5 m, including Tyrrhenian beach deposits at the base. The dipping of the layers towards the interior of the cave, the nature of the deposits and the absence of collapsed limestone blocks indicate that this first phase predates the opening of the roof. Level 22, described as a layer of sterile red clay, probably marks the intrusion of *terra rossa* from the slope above by open fractures in the roof. In the second infilling phase, from level 21 onwards, the roof of the cave opens gradually, seemingly without any major collapse. Clastic materials with a reddish clay matrix eroding from the slope entered the cavity through the growing hole in the roof and accumulated in the central part forming a large cone of clastic deposits leaning against the north wall.

The upper part of the main section (levels 1 to 11), cut through the upper two-thirds of the clastic deposits from the cone, was excavated in 1947. The excavation was continued in 1952 and 1953 from level 11 to level 51, representing 14 meters of deposits. The first 5 to 6 meters of infilling from the base are sterile or rather poor, while the last levels of the first phase (levels 27 to 23) have provided abundant remains attributed to the regional Mousterian facies referred to as “Pontinian” and industries from layers 27 and 26 were described as “denticulate Pontinian” [49–52].

Level 21 is known for its rich Aurignacian occupation [49, 53]. Several human remains have been unearthed in a Mousterian level of a secondary cavity, the Antro Obermaier or in the rich Aurignacian level of the central slope [54, 55].

Our fieldwork at Grotta del Fossellone in October 2012 was to refresh a small section to be sampled in April 2013 for luminescence dating. A small square (0.8 x 2m) was opened at the foot of the remaining upper section of A.C. Blanc through layers 1–21 and two small scaled sections were prepared, the lower one ranging from the base of layer 26 to layer 22 while only layer 21 was exposed in the upper one (Fig A in S1 File). The proposed correlation between 1947–1953 stratigraphy by Blanc and Segre [49] and the stratigraphy described for the 2012 section is reported in Table 1.

**Table 1:** Proposal of correlation between the 1947–1953 stratigraphy from [49] and the stratigraphy described for the 2012 section.

Description of deposits by Blanc and Segre	1947–1953 units	2012 stratigraphic units	Description of deposits	Lithic assemblages
Reddish clay with limestone angular fragments	F20 and upper	US 10	Reddish clay with limestone angular fragments	Not excavated
Brown incoherent loam [with limestone angular stones]	F21	US 11	Brown to brown-black clayey silt with more clayey orange lenses	Aurignacian
Red clay	F22	US 12	Homogeneous orange clay with a mid-height brown lens	Sterile
Grey-green sand	F23	US 13	Grey clay poor in siliceous granules filling gap between underlying limestone blocks	Mousterian
Dark gray clay with a stone pavement ("massicciata")	F24	US 14	Dark grey brecciated layer. Heavily altered limestone blocks with internal ferruginous concretions and underlined by manganese concretions are resting at the top	Mousterian
10 cm level called "contact" between 24 and 26	F25	US 15	Poorly sorted sand in a cemented grey to yellowish matrix	Mousterian
Sand with siliceous gravels	F26	US 16	Dark brown clayey silt, rich in charcoal. Reddish-brown strip at the top	Mousterian
		US 17	Brown-yellowish clayey sand rich in siliceous gravels	Mousterian
Conglomerate of small and medium size pebbles in a light gray cemented matrix	F27	US 18	Light gray cemented sandy layer with patches of stalagmitic floor	Not excavated

#### Dating

**Fig 3:**
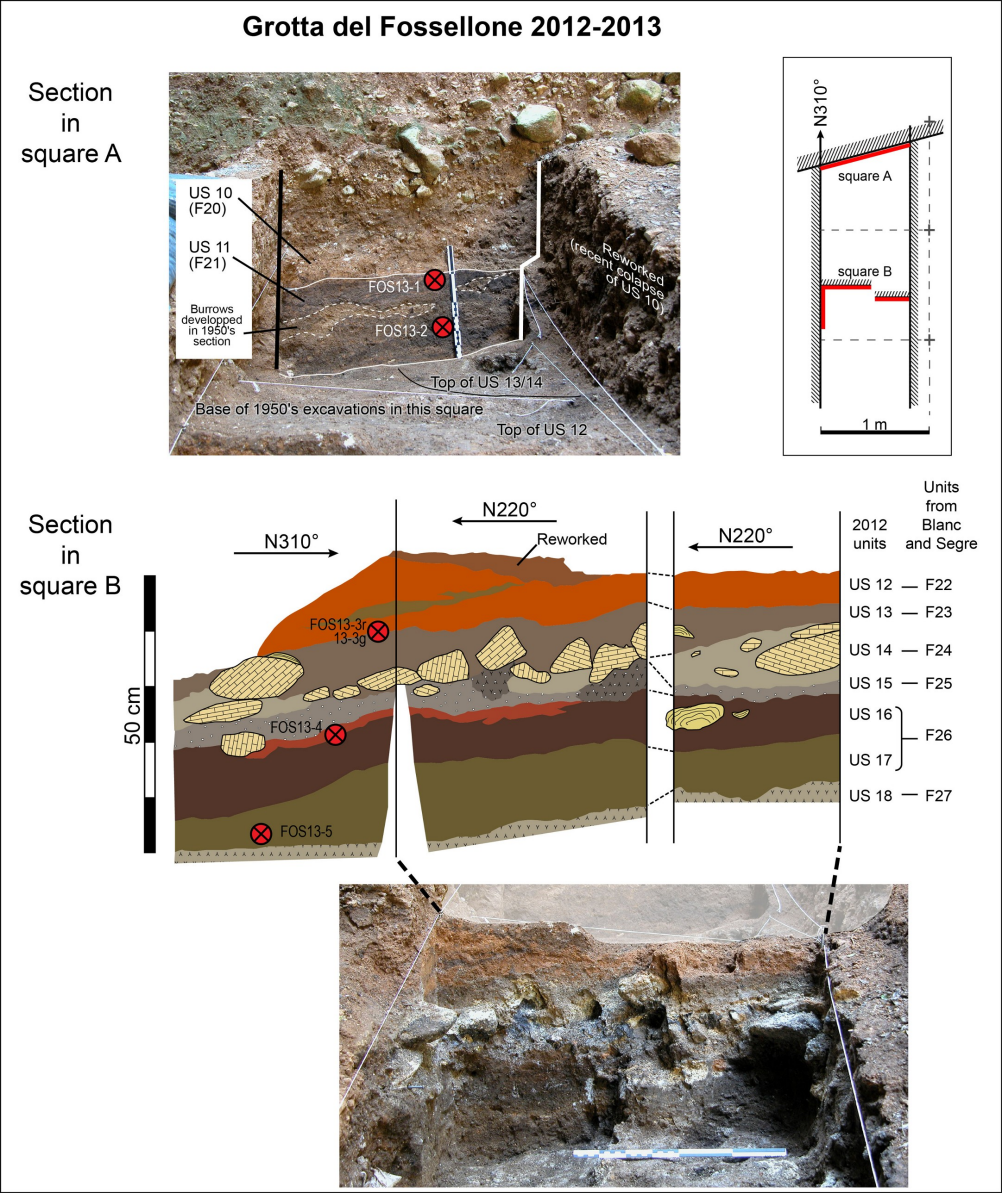
Grotta del Fossellone. Position of samples for luminescence dating in relation to the stratigraphic units defined in 2012.

Five samples were collected for optical dating from Fossellone Cave in 2013 (Fig 3 and Fig A in S1 file) and four ages were obtained from measurement of potassium-rich feldspar grains. Details about measurement of dose rates and equivalent doses are provided in the notes to Table 2. No age estimates could be obtained for FOS13-3 that was collected as a large intact sediment block and subsequently subsampled in the laboratory to separate samples from layers 22 (FOS13-3r) and 23 (FOS13-3g). All multigrain aliquots from these two sub-samples were saturated with respect to dose and further measurement of individual grains [29] resulted in a large spread of data suggestive of significant contamination by old and rotten bedrock material as well as bioturbation of sediment. It is, therefore, not possible to directly date layer 23 that contain the tools with adhesives, but must date to somewhere between ca.55 (Layer 26) and 40 (Layer 21) ka.

**Table 2:** Dose rate data, D_e_ values and luminescence ages for all samples in this study.

Sample code	Sample context		Field water content (%)	External dose rate (Gy/ka)			Internal dose rate (Gy/ka)^d^	Total dose rate (Gy/ka)^e^	D_e_ (Gy)^f^	Age (ka)^g^
	Layers (Blanc)	Layer (2012)		Gamma^a^	Beta^b^	Cosmic^c^				
FOS13-1	21	US 11	31.7	2.21 ± 0.09	2.23 ± 0.10	0.046	0.85 ± 0.07	5.34 ± 0.17	215 ± 14	40.3 ± 3.1
FOS13-2	21	US 11	33.6	2.10 ± 0.10	1.99 ± 0.09	0.046	0.85 ± 0.07	4.99 ± 0.16	206 ± 13	41.4 ± 3.1
FOS13-3r	22	US 12	35.0	1.80 ± 0.10	2.30 ± 0.12	0.046	0.85 ± 0.07	4.99 ± 0.17	—	—
FOS13-3g	23	US 13			2.17 ± 0.12	0.046	0.85 ± 0.07	4.87 ± 0.17	—	—
FOS13-4	26	US 16	34.0	1.21 ± 0.06	1.45 ± 0.07	0.046	0.85 ± 0.07	3.55 ± 0.14	200 ± 6	56.4 ± 2.6
FOS13-5	26	US 17	31.5	1.07 ± 0.05	1.46 ± 0.07	0.046	0.85 ± 0.07	3.43 ± 0.11	186 ± 8	54.2 ± 3.1

^**a**^ The gamma dose rates for all samples were measured directly at the point of sampling with a 2” in diameter NaI(Tl) detector. The dose rates were estimated using the “threshold” technique [56], which gives an estimate of the combined gamma dose rate from U and Th chains and from ^40^K. The detector was calibrated using the doped concrete blocks at Oxford [57].

^**b**^ The external beta dose rates for all 6 samples were made on sub-samples of dried, homogenized and powdered samples by GM-25-5 beta counting [58]. Dry dose rates calculated were adjusted for the water content and allowance was made for the effect of grain size and HF acid etching on beta dose attenuation.

^**c**^ The cosmic-ray dose rates were estimated from equations provided by [59], taking into account the burial depth of each sample (averaged over the entire period of burial), the density of sediment overburden (1.8 g/cm^3^), and the altitude (sea level) and longitude and latitude (41°13’26.29” N; 13°04’50.53” E) of Grotta del Fossellone. We assigned a relative uncertainty of ±15% to these dose rates to account for the systematic uncertainty in the primary cosmic-ray intensity [59].

^**d**^An internal beta dose rate was calculated by assuming internal ^40^K and ^87^Rb concentrations of 13 ± 1% and 400 ± 100 μg/g, respectively [60, 61]. These were converted to dose rates using the conversion factors of [62], and corrected for the absorbed dose fraction.

^e^ Mean ± total uncertainty (68% confidence interval), calculated as the quadratic sum of the random and systematic uncertainties.

^f^ Weighted mean ± 1σ uncertainty, calculated using the central age model. Individual D_e_ values were estimated from the 250°C MET-pIRIR signal [63] of 10 multi-grain aliquots containing ~300 K-feldspar grains, for each sample. An average residual dose of 6.8 ± 0.5 Gy was not subtracted.

^g^ The weighted mean of 8 measured fading rates was calculated (0.9 ± 0.3%), but ages were not corrected for fading

Seven teeth of Bos and Equus hydruntinus from layers 21, 23, 25, 26 and 27 excavated in the 1950’s were analyzed by Rainer Grün for ESR and U-series dating but did not yield significant results due to problems with calibrating the X-ray source used for irradiation. Thus layer 23 which contains the stone tools with organic residue studied in this paper can be dated only to the time interval between layer 26 and layer 21.

Layer 21, which provides a terminus ante quem for layer 23, is earlier than expected, so we provide below evidence that supports the early date.

#### The Aurignacian (layer 21)

Layer 21, defined in the literature as Aurignacian [49, 53], appears as a dark brown loam, sometimes interbedded with more clayey orange lenses, nearly 40 cm thick. During our fieldwork in 2012, this layer was worked frontally on less than 10 cm when partly refreshing the section left by A.C. Blanc. A few elements were collected (61 lithic pieces) and they confirm that it is the Aurignacian layer with in particular a broken end scraper, small bladelets and flakes from carinated end scrapers.

Some charcoal we have recovered in layer 21 was dated, as follows (Table 3):

**Table 3:** Radiocarbon dating on a charcoal sample from layer 21 by the oxford radiocarbon accelerator unit. The calibrated ages are given as 95.4% probability intervals.

AMS date, Laboratory no.	Date	+/-	Calibrated BP 95.4%
OxA-X-2507-43	33,950	750	40,762–36,946

The date is calibrated using the OxCal platform and the latest international calibration curve (IntCal 13; [64]. This determination was given an OxA-X code rather than OxA-as a warning due to a very low combustion yield (8%) which is linked with the sample being composed not of pure charcoal but of a mix of soil and charcoal. For this reason, the determination should be viewed as a minimum possible age. This age is statistically consistent with the optical ages for Layer 21 (Table 2). These ages overlap completely at 2-sigma confidence level (FOS13-1: 40.3 ± 3.1 ka and FOS13-2: 41.4 ± 3.1 ka).

For the past two decades, our knowledge of the chrono-cultural structure of the Aurignacian has been significantly enhanced and it is necessary to determine if this age agrees with the characteristics of the lithic and bone industry.

The complete study of this very rich assemblage exceeds the objectives of the present project so we focused on a sample in order to describe the bladelet production (Figs B-D in S1 File) as it was demonstrated that it discriminates between Aurignacian phases [65]. A very high frequency of carinated end scrapers was observed in this industry from layer 21 [66] in association with split-base points [49]. Aurignacian used thick flakes (around 3 cm thick) to shape carinated end scrapers [65, 67]. As it was almost impossible to produce such blanks by freehand percussion from the small flint pebbles so distinctive of the Latium region, Aurignacian flintknappers from Fossellone used the bipolar technique to produce them. Thick ovoid pebbles were specifically selected for this task. The aim was to open a large platform through the extraction of a thick longitudinal flake by bipolar percussion [68]. The platform was needed to shape carinated end scrapers from which bladelets were produced. The production of small bladelets from carinated end scrapers in association with split-base bone points is distinctive of the Early Aurignacian in France [65, 69–71]. The chronological model for the Aurignacian in Western Europe is now strongly supported by a wealth of radiocarbon measurements [72–80] allowing Banks et al. [81, 82] to propose chronological boundaries between stratigraphically hierarchized cultural entities: the 40.0–39.2 ka interval for the transition from Protoaurignacian to Early Aurignacian and the 37.0–36.5 ka interval for the transition from Early Aurignacian to Evolved Aurignacian. The industry of layer 21 from Fossellone is now confidently labelled Early Aurignacian and the ages (^14^C, luminescence) we obtained are in full agreement with the chronology described above. This type of Early Aurignacian is infrequent in Italy [83] but it was also identified at Riparo Mochi afterward [84]. Recent work confirms that at Mochi the Protoaurignacian (layer H) is followed by Early Aurignacian (layer G) [85, 86].

#### The Middle Paleolithic industry from layer 23 alpha

Layer 23 at Grotta del Fossellone is the latest Middle Paleolithic occupation in the stratigraphy of A.C. Blanc. It is followed by layer 22 which was described as sterile as confirmed by our own observations. The sample of the industry from layer 23 we selected was recovered in 1952 by A.C. Blanc in an irregular excavation grid area named α (alpha), adjacent to the south wall of the cave (Fig 2), which extend over almost 4 square meters. This sample of layer 23 alpha has never been published whereas the lithics from area γ (gamma) and β (beta) have been studied [50]. Our technological and typological analyses of this sample (459) are based on methods used in previous studies [87–89]. We present here the main results.

Most of the raw material was collected in the form of small pebbles, generally less than 5 cm length (Fig E in S1 File). Fossil or active beaches were the sources of these raw materials [90] but they are now buried under Holocene colluvial deposits. Fine-grained flint is dominant (75.8%) but coarse flint or less silicified materials (chert, silicified limestone) were also selected. Blanks were produced through four types of chaîne opératoire (Figs F-G in S1 File). Several cores were reduced using the bipolar technique [68] and a third of the flakes were retouched. A good number of flakes with two opposed impact points and non-conchoidal fracture display use of the common bipolar on anvil technique, not the “Pontinian” variant so distinctive of the industry from Sant’Agostino (Fig H in S1 File). Levallois debitage is unambiguously identified. It is poorly represented (Table 4) but three-quarter of the Levallois flakes have been retouched, the highest tool/debitage ratio. Flakes with scars of successive series of unidirectional parallel removals, which can come from the same platform, or an opposed or orthogonal one (together with cores characterized by a diversity of scars patterns) correspond to a simple scheme to produce regular blanks often present in Middle Paleolithic industries [87]. The amount of pseudo-Levallois and débordant flakes including typical pseudo-Levallois points is a direct evidence of discoid production [91, 92].

**Table 4:** Fossellone layer 23α. **Counts of debitage and cores**. Broken flakes without a platform, flakes <1.5 cm and chunks are excluded. Flakes from tool making or tool reworking of any size are included.

Flakes	N	%	Cores	N	%
Levallois flakes	4	2.1	Levallois cores	4	8.2
Pseudo-Levallois and débordant flakes	25	13.0	Discoidal cores	5	10.2
Bipolar flakes	33	17.2	Bipolar cores	14	28.6
Unidirectional flakes	29	15.1	Unidirectional cores	10	20.4
All other flakes	101	52.6	All other cores	16	32.7
Total	192	100.0	Total	49	100.0

Scrapers dominate the tool-kit (79.4%). The remaining part comprises (in decreasing frequency) irregularly retouched flakes, denticulates, Mousterian points, end scrapers and retouched notches. Frequently (25/161 = 15.5% of retouched tools) older altered or patinated blanks (retouched tools, debitage) were collected and retouched again. Recycling is not uncommon in Middle Paleolithic industries [93–95], but it is especially developed in this assemblage. Scaled pieces are a minor component of the industry. Only two flakes coming from scaled pieces have been retouched.

**Fig 4:**
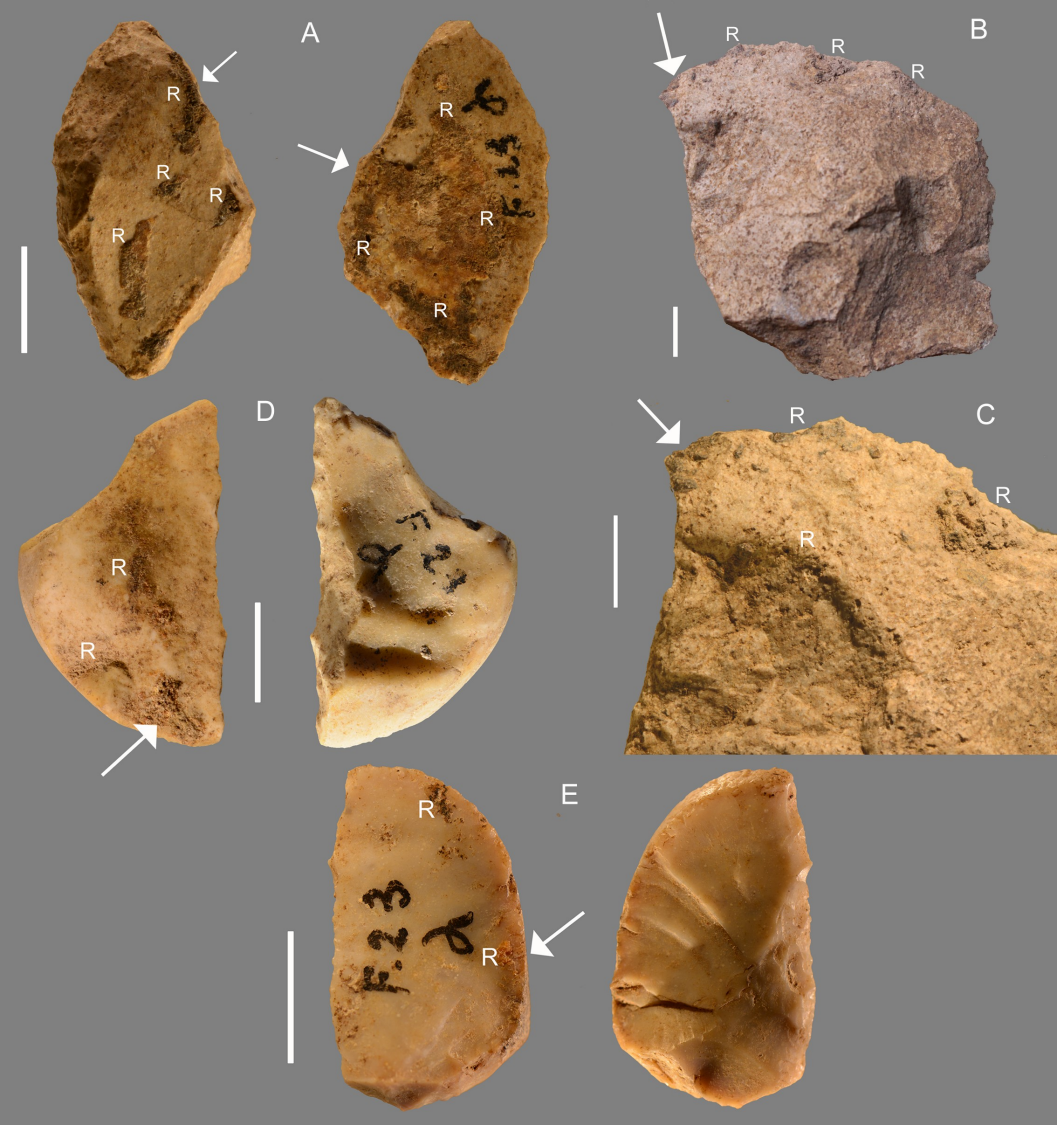
Grotta del Fossellone, layer 23 alpha. **Stone tools with analyzed residue**. The white arrow indicates the analyzed sample; R indicates macroscopically visible residue. All of flint except (B). (A) F1, side scraper. (B) F4, unretouched flake, silicified limestone, oriented according to the debitage axis; (C) detail of F4. (D) F5, transverse scraper, oriented according to the morphological axis; (E) F3, side scraper. Significant molecular markers of organic materials were found in all these pieces. Four other artifacts that gave no significant results are illustrated in Fig P in S2 File. Scale bar = 1 cm.

The four pieces with verified residues from layer 23α are typologically and technologically variable (Fig 4). The most normalized flakes such as Levallois flakes or pseudo-Levallois points are not present in this set.

### Grotta di Sant’Agostino

**Fig 5:**
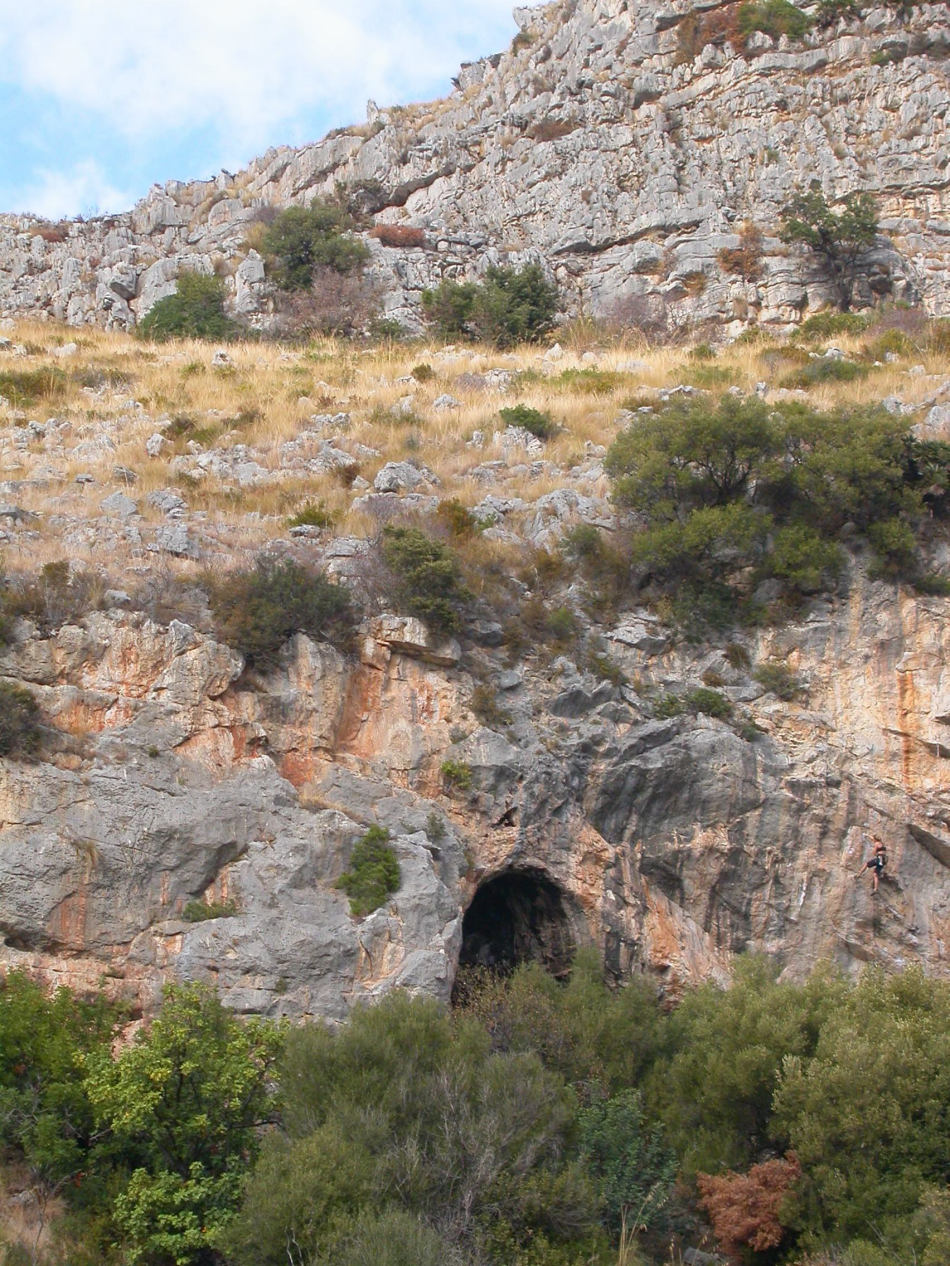
View of Grotta di Sant’Agostino.

This cave, 16 by 18 m and 50 m asl, was excavated in 1947 and 1948 by the Institute of Human Paleontology in Rome, the excavations were directed by E. Tongiorgi [96] (Fig 5). One shallow excavation trench, only 50 cm deep, was opened in the front part of the cave; it contained abundant fauna and only few artifacts. The main excavation trench in the internal part of the cave had a maximum thickness of 2.5 m and contained an abundant Mousterian lithic industry, defined as “Pontinian” (essentially a Mousterian on small pebbles) by A.C. Blanc in 1937 for assemblages in the Latium area.

The deposit in the main trench consisted of more or less concreted sand resting on a stalagmite. It was divided in five levels (A, A1-A4). The surface level (A) was disturbed due to modern use of the cave as a sheep pen and contained a mixture of Middle and Upper Paleolithic artifacts. Levels A1-A4 were excavated over an area of about 5.75 to 3 square meters, decreasing by depth. They could not be correlated to the deposit in the front of the cave; the level boundaries were not clearly distinct, but the levels were kept separate to divide the materials according to depth. A study of the large lithic and faunal assemblage was published by Carlo Tozzi in 1970 [96].

#### Dating

The deposit rests on a stalagmite, dated by U-series to 112 ± 14 ka and 120 ± 15 ka [97]. The ESR ages for levels 1 to 3 were done on tooth enamel. Tooth samples of known provenience were obtained from the Italian Institute of Human Paleontology in Rome. The external dose rate was obtained by using a portable gamma-ray spectrometer. Since the trenches had been filled up and sections were no longer visible, the measurements were made in holes cut into the deposit approximately at the level at which teeth had been obtained [97]. ESR ages for layer A1 was 43 ± 9 ka, for A2 was 53 ± 7 and for A3 was 54 ± 11 ka. All these ages represent an average of multiple individual estimates [97].

Our 2011 visit to the cave confirmed that the location of trenches can no longer be determined without extensive re-excavation of the cave floor (Fig I in S1 File). Since suitable sediment samples cannot be obtained, OSL dating was ruled out. Radiocarbon dating of two animal bones from level A1 was attempted by the Oxford Laboratory but failed as the bones did not preserve collagen. A unique fragment of a *Mytilus* shell found by us in level A1 provided a final Upper Paleolithic date (OxA-27,399: 11830 ± 45 BP (13760–13550 cal BP) and was clearly intrusive from level A above. In fact, our analysis of the A1 assemblage indicated that 18 stone artifacts were Upper Paleolithic, including some Epigravettian.

#### The A1 lithic assemblage

Of the four levels with Mousterian lithics, level A1 has yielded the larger number of artifacts (3138 including all flake fragments and chunks [96]. The industry shows substantial homogeneity without significant differences between levels. We decided to do a systematic technological and typological analysis of level A1 only and we present here the main results of our study.

Level A1 was excavated on 5.75 square meters and was 30 cm thick. The total lithic assemblage of Sant’Agostino layer A1 is large (Table 5). The debitage is relatively abundant (about 5 flakes per core). The retouched tools (n = 763) are dominated by side scrapers (66%). Flint pebbles, generally smaller than 5–6 cm, are the dominant raw material (1561/1608 = 97%). The pebbles are lenticular in cross-section and of very fine-grained flint. Their water-worn cortex and their regular shape indicate that the sources were beaches now buried by late Pleistocene and Holocene sands and alluvial deposits [90].

**Table 5:** Sant’Agostino layer A1. **Counts of debitage and cores**. Broken flakes without a platform, flakes <1.5 cm and chunks are excluded. Tool retouch flakes of any size are included.

Flakes	N	%	Cores	N	%
Flat flakes	150	9.3	Cores with flat removals	21	6.4
Levallois flakes	22	1.4	Levallois cores	22	6.7
Bipolar flakes	8	0.5	Bipolar cores	6	1.8
All other flakes by direct percussion	1428	88.8	All other cores by direct percussion	278	85.0
Total	1608	100.0	Total	327	100.0

Three types of chaînes opératoires (i.e. reduction sequences) were used to produce blanks (**Table 6**). The most characteristic component is a particular variant of the bipolar technique [88]. The bipolar technique (or hammer and anvil technique) consists in resting a pebble (or a block or a flake) on anvil and striking it with a percussor (Fig H in S1 File). Flakes produced with this technique are characterized by a non-conchoidal fracture, a flat ventral face, a crushed or concave or no bulb of percussion, no measurable platform and an opposing bulb or shattering at the distal end.

**Table 6:** Sant’Agostino layer A1. **Blanks of retouched pieces**. Indeterminate flakes and indeterminate blanks are excluded.

Categories	N	%
Flakes by direct percussion	363	61.4
Flat flakes	160	27.1
Levallois flakes	8	1.4
Bipolar flakes	4	0.7
Cores by direct percussion	12	2.0
Cores with flat removals	36	6.1
Levallois cores	1	0.2
Bipolar cores	1	0.2
Pebbles	6	1.0
Total	591	100.0

However, the flakes of Sant’Agostino do not show features expected on bipolar flakes. Experiments show that the lack of opposing bulb or other traces at the distal end were probably either due either to the use of a soft anvil (e.g. wood or soft limestone) or more simply by resting the pebble on the ground and maintaining it in a vertical position [98]. This very specific variant of the bipolar technique (Figs H: B in S1 File) is particularly apt to the use of small pebbles, to initiate a flaking sequence or to obtain cortical and partly cortical flakes with an undamaged distal edge, a significant feature in very small products. This technique is typical of the above mentioned “Pontinian” assemblages [49–52, 88–90, 99]. We call these flakes “flat flakes” (Figs H: C in S1 File). Table 6 shows that 27% of retouched pieces were made on flat flakes and 6% on cores with flat scars.

**Fig 6:**
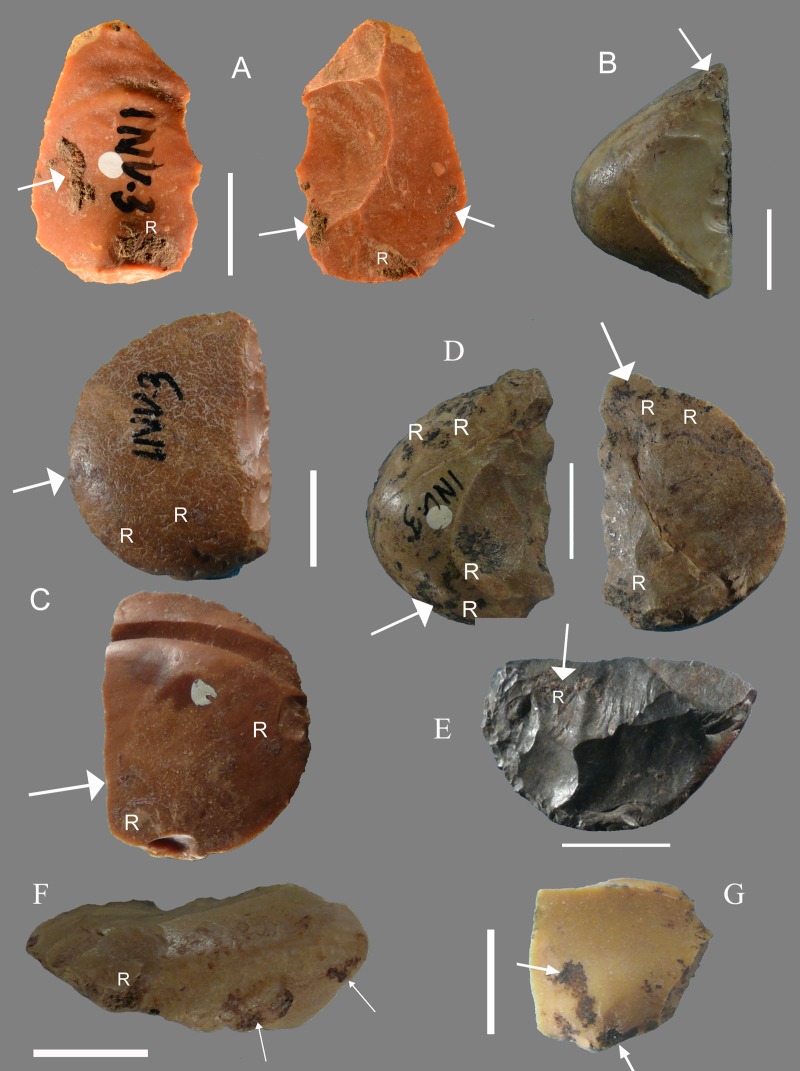
Stone tools with analyzed residue from Sant’Agostino cave, layer A1, all tools are of flint. The white arrow indicates the analyzed sample; R indicates macroscopically visible residue. (A) Levallois flake no. L2; (B) transverse scraper no. 362, oriented according to the morphological axis; (C) Side scraper no. 114; (D) Side scraper no. 258; (E) Scraper no. 211; (F) Transverse scraper no. 268; (G) Small unretouched flake, catalogue no. M1. All pieces gave significant results with the exception of (G). Scale bar = 1 cm.

The Levallois technology is very little represented and the classical bipolar modality even less (Tables 5 and 6). A third and much more common reduction sequence is documented by non-Levallois cores and flakes with series of unidirectional, bidirectional or multidirectional removals by direct percussion, without any special preparation of the debitage surface or shaping of the core. Flakes so produced have unidirectional, orthogonal or convergent scars on the dorsal face and cortical or simply prepared platforms (Figs S10E in S1 File). All three reduction sequences are represented among the tools with hafting adhesive (Fig 6). Nos.114 and 362 are on flat flakes, L2 is on a Levallois flake, no. 258 is a partly cortical ordinary flake, no. 211 is on an undetermined flake (missing the platform).

#### Chemical analysis of Fossellone and Sant’Agostino Caves

Sampling for GC/MS analysis. All artifacts from layer Fossellone layer 23 alpha and from Sant’Agostino layer A1 were examined for macroscopically visible residues. Seven artifacts at Fossellone were selected from a total of 360 retouched and debitage pieces, excluding cores, bipolar cores and scaled pieces. One piece was found among the sample of Fossellone layer 23 gamma at the Pigorini Museum. From the Sant’Agostino assemblage of layer A1 seven artifacts were selected with macroscopically visible residue; the total number of examined retouched pieces and flakes is 2140, excluding cores, pebbles, scaled pieces and undetermined blanks. Amorphous organic material was sampled by light scraping with stainless steel scalpels. The artifact description and weight of sample (in mg) for each of the Fossellone and Sant’Agostino selected pieces are provided in Table 7. Several samples from Fossellone and one from Sant’Agostino weighed more than 0.1 mg, while two from Fossellone and six out of seven from Sant’Agostino weighted less than 0.1 mg. In spite of the reduced amount of sample, our procedure allows for detecting nanograms of organic material inside samples, and environmental and procedure blanks are run periodically, thus ensuring the representativeness of the results obtained on the microsamples.

**Table 7:** Description of the artifacts and weight of the analyzed samples.

Sample code	Sample weight [mg]	Description	Photo
F1	< 0.1	Fossellone, layer 23 alpha. Flint side scraper	Fig 4A
F2	< 0.1	Fossellone, layer 23 alpha. Unretouched flint flake	Fig P: A in S2 File
F3	1.2	Fossellone, layer 23 alpha. Flint side scraper	Fig 4E
F4	5.0	Fossellone, layer 23 alpha. Unretouched flake of silicified limestone	Fig 4B and 4C
F5	4.9	Fossellone layer 23 alpha. Flint transverse scraper	Fig 4D
F6	5.2	Fossellone layer 23 alpha. Flint broken scraper	Fig P: B in S2 File
F7	1.5	Fossellone, layer 23 alpha. Denticulate, flint	Fig P: C in S2 File
F8s	4.0	Fossellone section, for sample OSL3, upper red band, sediment	Fig 3
F9s	4.0	Fossellone section, for sample OSL3, lower gray band, sediment	Fig 3
F10	1.4	Fossellone layer 23 gamma, Pigorini Museum no. 179081, side scraper	Fig P: D in S2 File
AGO 1	< 0.1	Sant’Agostino, level A1. Unretouched flint flake, M1	Fig 6G
AGO 2	< 0.1	Sant’Agostino, level A1. Flint side scraper no. 114	Fig 6C
AGO 3	< 0.1	Sant’Agostino, level A1. Flint scraper no. 211	Fig 6E
AGO 4	< 0.1	Sant’Agostino, level A1. Flint side scraper no. 258	Fig 6D
AGO 5	< 0.1	Sant’Agostino, level A1. Flint transverse scraper no. 268	Fig 6F
AGO 6	< 0.1	Sant’Agostino, level A1. Flint transverse scraper no. 362	Fig 6B
AGO 10	0.9	Sant’Agostino, level A1. Flint Levallois flake	Fig 6A
B1	2.3	Sant’Agostino, level A1. Medial fragment of radius-ulna of *Cervus elaphus*	Not illustrated
B2	1.5	Sant’Agostino, level A1. *Bos* Calcaneum	Not illustrated

The sampled material was subjected to a combined analytical procedure [100–101] for the identification of lipids, waxes, resinous, proteinaceous and saccharide materials in the same micro-sample. Further details on procedures and instrumentation are provided in S2 File.

## Results

Neither the analysis of the proteinaceous nor of the polysaccharide fraction yielded results above the detection limits of the protocol, thus suggesting the absence of protein- or sugar-based materials in the samples.

The results of the analyses of the lipid-resinous fraction, which is performed after saponification of the relevant fraction of the original sample, and extraction of neutral components in hexane and acidic components in diethyl ether (after acidification) are presented below. Note that both fractions were admixed, dried, derivatized to obtain trimethylsilyl (TMS) derivatives and injected in the same analytical run to pre-concentrate the analytes of interest [100–101]. The identified organic compounds are summarized in Table 8.

**Table 8:** List of compounds identified in the lipid-resinous fraction of the analyzed samples (the columns relative to the analytical blanks are highlighted in gray). Acids and alcohols are identified as their TMS derivatives.

N	compounds	F1	F2	F3	F4	F5	F6	F7	F8s	F9	F10	AGO1	AGO2	AGO3	AGO4	AGO5	AGO6	AGO10	B1	B2
1	decanoic acid	x	x		x	x	x	x	x		x									
2	hexanedioic acid	x	x		x	x														
3	2-hydroxybenzoic acid	x	x		x	x														
4	4-methoxy-benzoic acid	x	x																	
5	undecanoic acid	x	x																	
6	dodecanol		x				x		x		x									
7	heptanedioic acid	x	x																	
8	4-hydroxybenzoic acid	x	x	x			x	x			x									
9	dodecanoic acid	x	x	x	x	x	x	x	x		x	x	x	x	x	x	x	x	x	x
10	tridecanol		x																	
11	tetradecanol	x	x		x	x	x				x									
12	4-hydroxy-hydrocinnamic acid	x	x	x	x	x	x	x	x	x	x		x	x	x	x	x	x		
13	nonanedioic acid (azelaic acid)	x	x		x	x	x		x		x		x	x	x	x	x	x	x	
14	tetradecanoic acid (myristic acid)	x	x	x	x	x	x	x		x	x	x	x	x	x	x	x	x	x	x
15	pentadecanol				x	x	x				x									
16	decanedioic acid (sebacic acid)	x	x	x	x	x	x				x									
17	pentadecanoic acid (• branched)	x	x	x	x	x	x	x	x		x									
18	hexadecanol	x	x	x	x	x	x	x	x		x	x	x	x	x	x	x	x	x	x
19	undecanedioic acid	x	x	x		x	x				x									
20	9-trans-hexadecenoic acid (palmitelaidic acid)	x	x	x	x	x	x		x		x	x	x	x	x	x	x	x	x	x
21	hexadecanoic acid (palmitic acid, • branched)	x	x	x	x	x	x	x	x	x	x	x	x	x	x	x	x	x	x	x
22	heptadecanol					x														
23	heptadecanoic acid (• branched)	x	x	x	x	x	x	x	x		x	x	x	x	x	x	x	x	x	x
24	octadecanol	x	x	x	x	x	x	x	x	x	x	x	x	x	x	x	x	x	x	x
25	docosane	x				x		x												
26	9,12-octadecadienoic acid (linoleic acid)	x	x	x	x			x										x	x	x
27	octadec-9-enoic acid (oleic acid)	x	x	x	x	x	x	x	x		x	x	x	x	x	x	x	x	x	x
28	octadecanoic acid (stearic acid)	x	x	x	x	x	x	x	x	x	x	x	x	x	x	x	x	x	x	x
29	tricosane	x				x		x												
30	14-hydroxyhexadecanoic acid	x																		
31	15-hydroxyhexadecanoic acid	x																		
32	nonadecanoic acid	x				x														
33	eicosanol	x	x	x	x	x	x				x									
34	tetracosane	x				x		x												
35	dehydroabietic acid (DHA)	x		x	x	x							x	x	x	x	x	x	x	x
36	eicosanoic acid	x	x	x	x	x	x		x		x		x	x	x	x	x	x		
37	pentacosane	x						x		x										
38	heneicosanol	x	x																	
39	9,10- dihydroxyoctadecanoic acid isomer	x																		
40	9,10-dihydroxyoctadecanoic acid isomer	x																		
41	hexacosane	x	x			x		x												
42	7-oxo-dehydroabietic acid (7-oxo-DHA)	x		x	x	x							x	x	x	x	x	x		
43	docosenoic acid (erucic acid)	x	x		x		x													
44	docosanoic acid	x	x			x							x		x		x			
45	heptacosane	x						x												
46	tricosanoic acid	x																		
47	tetracosanol	x				x														
48	octacosane	x						x												
49	tetracosanoic acid	x	x			x							x		x		x			
50	nonacosane	x						x												
51	triacontane	x						x												
52	hexacosanoic acid	x																		
53	hentriacontane	x						x												
54	cholesterol	x	x	x					x		x	x	x	x	x	x	x	x	x	
55	dotriacontane	x						x												
56	β-sitosterol	x	x																	
57	Squalene											x	x	x	x	x	x	x	x	x
58	methyl-dehydroabietate															x		x		

**Fig 7:**
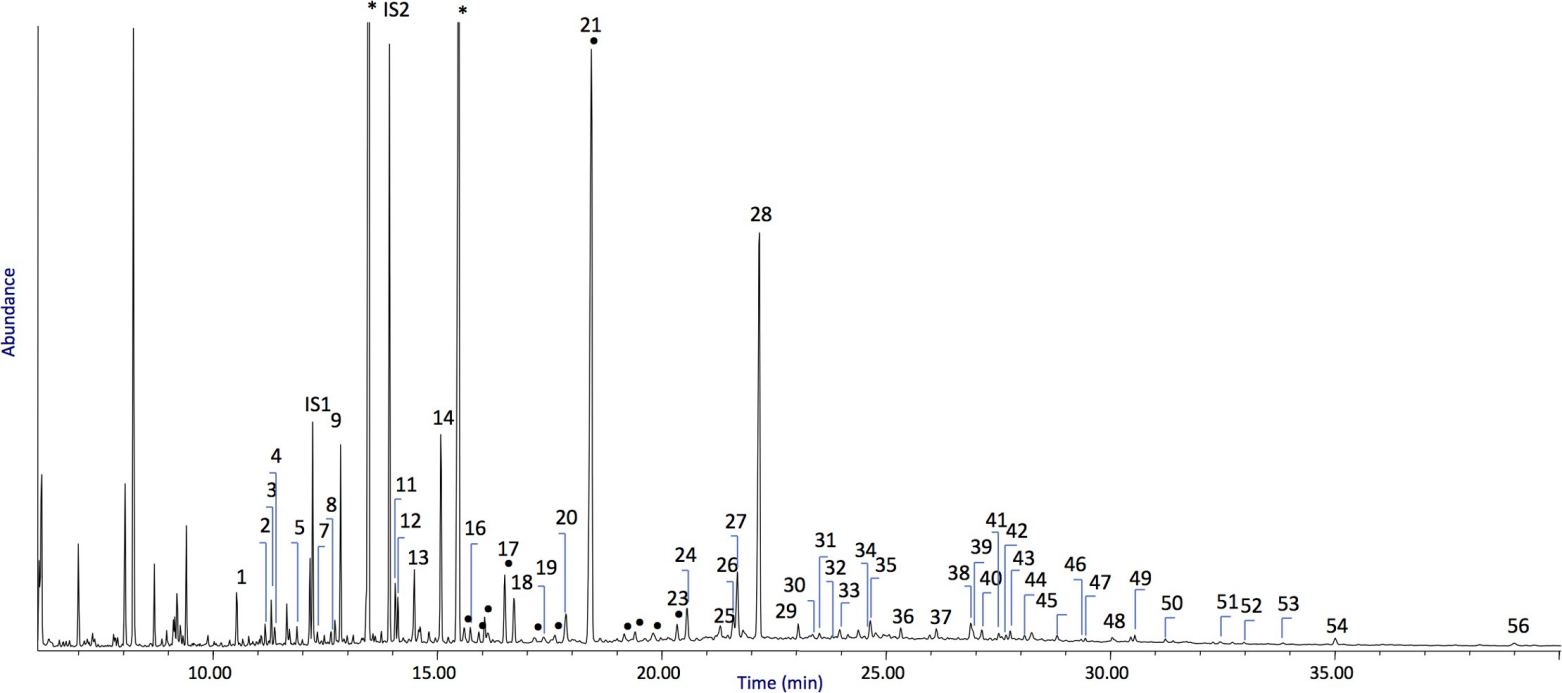
Chromatographic profile obtained for lipid-resinous fraction of sample F1. The numbers refer to Table 8. IS1 = hexadecane, IS2 = tridecanoic acid, •: branched fatty acids, *: phthalate contamination.

**Fig 8:**
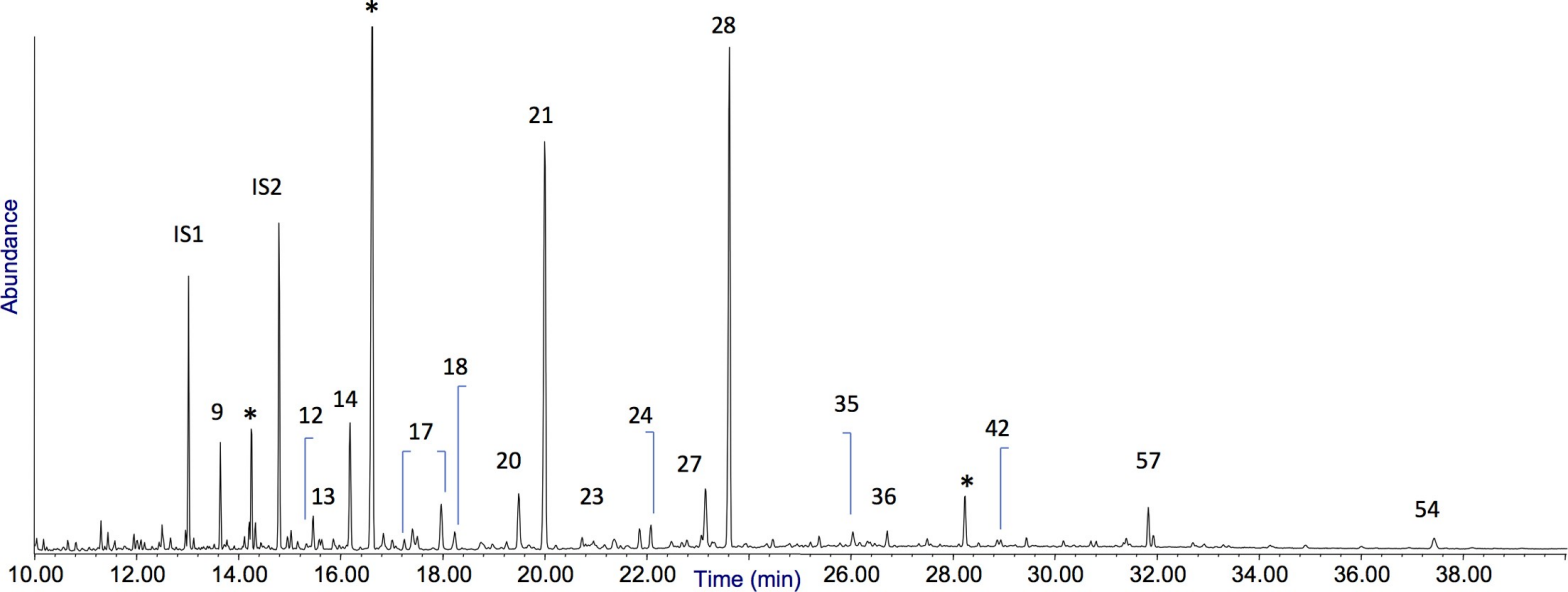
Chromatographic profile obtained for lipid-resinous fraction of sample AGO4 no. 258. The numbers refer to Table 8. IS1 = hexadecane, IS2 = tridecanoic acid, *: phthalate contamination.

**Fig 9:**
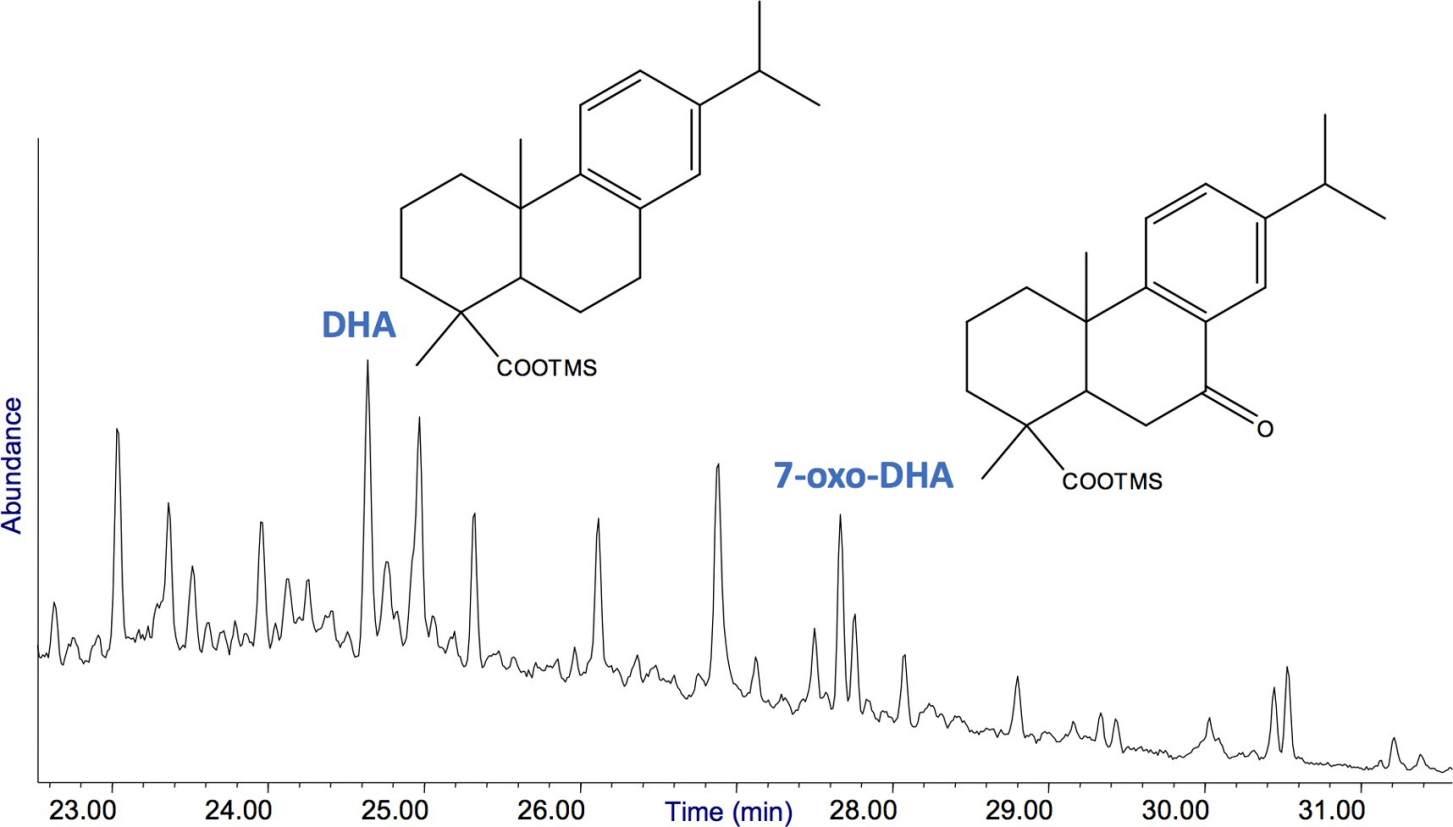
Inset of the GC/MS profile of sample AGO 10 (the Levallois flake) highlighting the peaks due to methyl-dehydroabietate (Me-DHA), dehydroabietic acid (DHA) and 7-oxo-dehydroabietic acid (7-oxo-DHA). The full chromatogram is presented in Fig K in S2 File.

Chromatograms of environmental blanks are shown in Figs A-C of the S2 File for Fossellone and Sant’Agostino. Relevant chromatograms from Fossellone are in Fig 7 and Figs D-F in S2 File. For Sant’Agostino relevant chromatograms are in Figs 8 and 9 and in Figs G-K in S2 File. The profiles of three artifacts from Fossellone (F2, F6, F10) that gave no significant results and one from Sant’Agostino (AGO 1) equally without results are in Figs L-O in S2 file.

### Environmental blanks (Figs A-C in S2 File)

To exclude contamination due to burial conditions, soil samples from a stratigraphic section were collected as environmental blanks and analyzed. This was the case for the Fossellone samples. No sections were available for Sant’Agostino; however, some bones still had some adhering sediment. Thus, we selected two bones from level A1 and we analyzed the adhering sediment as the reference environmental blank.

By comparing the results obtained for the samples and the environmental blanks, we assessed the contribution of soil contamination. The intensity of the peak assigned to the internal standards IS2 (tridecanoic acid, internal standard for derivatization) was used, together with samples weight and the volume of the final injected solution to normalize the relative intensities of the other peaks in the chromatograms for proper comparison amongst the batches and to draw sound conclusions with respect to the presence of hafting material on the artifacts.

Fig A in S2 File shows the profile obtained for the sample of sediment belonging to Fossellone. For the sediment on bone samples of Sant’Agostino, the chromatograms are presented in Figs B-C in S2 File. Phthalates (marked with * in the chromatograms) are ubiquitous contaminants in the environment and were detected both in the blanks and in the samples; squalene is a contaminant due to manipulation of the artifacts and was detected both in the blanks and in the samples from Sant’Agostino.

### Chromatograms of Fossellone cave (Fig 7 and Figs D-F in S2 File)

Peak assignment are provided in Table 8.

The main acidic components in all the Fossellone samples were long chain (10–20 carbon atoms) linear monocarboxylic acids. The acids were determined in significant amount, both above the detection limits of the analytical procedure and above the environmental blank level assessed in sediment samples, as can be seen by comparing the intensities of the chromatograms acquired for the residues and for the sediment, pointing to the presence of a lipid of plant origin (non-siccative). Branched fatty acids and cholesterol were both present in the sediment and in the samples in similar amounts and were thus not considered for further interpretation on hafting materials.

Lignoceric acid (the linear monocarboxylic acid with 24 carbon atoms) was present in samples F1, F2, and F5 only (chromatograms in Fig 7 and Figs L and F in S2 File), and hexacosanoic acid (C26:0), also called cerotic acid, was identified in sample F1. The most abundant acids were palmitic (hexadecanoic acid, C16:0) and stearic acid (octadecanoic acid, C18:0). In all the samples, long chain alcohols (12–20 carbon atoms) were identified in different relative amounts. The presence of long-chain monocarboxylic fatty acids along with long chain alcohols and alkanes in significant amount with respect to the blank indicated the presence of plant material, including glycerolipids and waxes [102].

Moreover, in sample F1 the presence of long chain fatty acids (C10-C26) together with long chain alcohols (C12-C24) and (ω-1)-hydroxy and (ω-2)-hydroxy-hexadecanoic acids (peaks #30 and #31 respectively) points to the presence of beeswax [103]. As expected, long chain fatty acids (C10-C26) together with long chain alcohols (C12-C24) are also present, whose profile is typical of a heated (by accident or on purpose) beeswax, admixed with a glycerolipid of plant origin [104,105].

The terpenic fraction of samples F1, F3, F4 and F5 is relevant. The detection of diterpenes as dehydroabietic (#35) and 7-oxo-dehydroabietic (#42) highlights that the sample contained a material collected from a resin exuded from plants of the Pinaceae family [106]. Diterpenes more oxidized than 7-oxo-dehydroabietic were not detected in the samples.

In the case of sample F1, both beeswax and Pinaceae resin were identified, consistent to the recent observation that the addition of beeswax to a diterpenoid resin increases the adhesive properties of the mixture with respect to the single components [107]. Use of beeswax as a cement is documented ethnographically by a number of hatchet heads in Australian museums [12] and on points of the Final Paleolithic in Germany [108].

### Chromatograms of Sant’Agostino (Figs 8 and 9 and Figs G-K in S2 File)

All the profiles show the presence of a number of fatty acids (saturated and unsaturated). The saturated and unsaturated fatty acids were ascribed to a contamination from the soil, as proved by comparison with their intensities in the environmental blanks (chromatograms in Fig B-C in S2 File). Samples AGO2-6 and AGO10 contained diterpenes in relevant amount (dehydroabietic acid and 7-oxo-dehydroabietic, peaks #35 and #42, respectively, highlighted in the chromatograms and whose structure is presented in Fig 9), thus proving the presence of a resin exuded from plants of the Pinaceae in 6 out of seven samples. The detection of methyl-dehydroabietate (peak #58) in samples AGO5 and AGO10 might suggest that the resin was heated in the presence of wood, which produces methanol causing in turn the methylation of dehydroabietic acid [106, 109]. The absence of retene or other highly aromatized diterpenes rules out the possibility that the hafting material was pitch [110].

## Discussion

The analytical results, summarized in Table 9, show that the residues in Fossellone cave derive from plant lipids (oil and/or waxes) in some cases mixed with Pinaceae resin possibly to improve the adhesive properties of the resin. In one sample, beeswax mixed with the resin was detected. In Sant’Agostino cave samples, the material employed for hafting is Pinaceae resin, which was, at least in two cases (AGO 5, scraper no. 268 and AGO 10 Levallois flake) probably heated in the presence of wood before application. This should not be a surprise since resin is a thermoplastic material. Although resin is viscous (sticky) when it exudes from the tree, it dries exposed to the air. Since the sites are two caves where debitage, retouching and domestic activities were carried out it is likely that resin was collected at some distance from the cave. Then warming of the resin was needed. Ethnographic evidence indicates that resin was generally warmed and softened by moderate heat, such as holding the collected resin near a small fire or on its embers. Once softened the resin is pliable and can be molded and pushed in position in the haft and around the stone tool with a pointed stick [23]. The resin then sets again and hardens as it cools down, keeping the stone in place [12]. The hardened resin can be reheated and melted for re-hafting.

**Table 9:** Summary of results of the gas-chromatographic analyses.

Samples	Description	Glycerolipids (including plant waxes)	Beeswax	Pinaceae resin
F1	Fossellone layer 23 alpha, side scraper	X	X	X
F2	Fossellone layer 23 alpha, unretouched flake	X	-	-
F3	Fossellone layer 23 alpha, side scraper	X	-	X
F4	Fossellone layer 23 alpha, flake of silicified limestone	X	-	X
F5	Fossellone layer 23 alpha, transverse scraper	X	-	X
F6	Fossellone layer 23 alpha, broken scraper	X	-	-
F7	Fossellone layer 23 alpha, denticulate	-	-	-
F10	Fossellone layer 23 gamma, Pigorini Museum no. 179081, side scraper	X	-	-
F8 sediment	FOS13-OSL3, upper red band, sediment	-	-	-
F9 sediment	FOS13-OSL3, lower gray band, sediment	-	-	-
AGO1	Sant’Agostino level A1, unretouched flake M1	Blank level	-	-
AG02	Sant’Agostino level A1, side scraper no.114	Blank level	-	X
AGO3	Sant’Agostino level A1, scraper no. 211	Blank level	-	X
AGO4	Sant’Agostino level A1, flint side scraper no. 258	Blank level	-	X
AGO5	Sant’Agostino level A1 flint transverse scraper no. 268	Blank level	-	X
AGO6	Sant’Agostino level A1, flint transverse scraper no. 362	Blank level	-	X
AGO10	Sant’Agostino level A1, Flint Levallois flake no. L2	Blank level	-	X
B1 adhering sediment	Sant’Agostino, level A1, medial fragment of radio-ulna of *Cervus elaphus*	Blank level	-	-
B2 adhering sediment	Sant’Agostino, level A1, *Bos* calcaneum	Blank level	-	-

We call attention to the fact that even an accurate examination under the microscope does not allow the unambiguous detection of hafting residues, since the aspect of the residues on the pieces were all very similar in spite of the different composition. FTIR or other spectroscopic techniques might provide more insights on the nature of the residues, but would not allow a reliable comparison with the environmental blanks (sediments from section at Fossellone or adhering to bones at Sant’Agostino) due to their lower sensitivity especially towards minor components in a complex mixture. Only the thorough application of micro-destructive techniques, in parallel with the analysis of environmental blanks, allows for discriminating the residues on artifacts between actual hafting material and the site sediment and for the taxonomic identification of the resinous materials.

### Incidental deposition

On several pieces residue traces are present on the proximal and lateral (unretouched) edge. This is the case of Fossellone F1 (Fig 4A) F5 (Fig 4D) F3 (Fig 4E) and for Sant’Agostino the Levallois flake (Fig 6A), side scraper 114 (Fig 6C), and scraper 211 (Fig 6E). The latter has a residue strictly parallel to the working edge. Three pieces require a different interpretation. On one piece (Fig 6B, scraper 362 of Sant’Agostino) the organic residue is all along the scraping edge suggesting that the tool was used for collecting softened resin during dehafting. Alternatively, the scraper might possibly have been used for collecting resin from a trunk of a conifer tree but since the tool was abandoned after its last use in a habitation site, i.e. a cave away from trees, this hypothesis is less plausible. On F4 (Fig 4B) the residue is present mostly on the distal edge. This in an unretouched flake oriented according to the debitage axis. Three edges could have been used so the residue orientation does not inform us on the hafted area. There is a possible thermal scar on its right side. On Sant’Agostino side scraper 258 (Fig 6D) the residue traces are concentrated on proximal and distal edges but some stains are dispersed on the dorsal or the ventral face of the piece. This could be taken as evidence of syn-or post-depositional contamination thus excluding the hypothesis of hafting. However this is not the case for our pieces because (1) there is no evidence of resin in the surrounding sediment; (2) if the artifacts with Pinaceae residue were in or near a fire containing embers of Pinaceae wood, dripping resin could have transferred to the tool surface [111–112]. However, with the possible exception of flake F4, none of the other artifacts in this study have been heat treated: there are no changes in color, nor fractures or thermal scars due to heat.

There is instead clear evidence that incomplete preservation of the adhesive producing this pattern of dispersed stains occurred at several sites where the organic residue was identified. At Umm el Tlel some Levallois flakes show large uniform stains but others have stains with a dispersed pattern ([39]: Figs 3, 4 and 5). On 30 flakes the amount preserved was insufficient for chemical analysis-which means that it occurred as small spots ([39]: p. 69 note 14). At Hummal small black stains of bitumen occur on a Mousterian point and on a Levallois flake ([40]: fig. 2: 1, 3).

### Conclusions

On most pieces the localization of the identified residue is consistent with lateral-proximal hafting. The ten pieces with identified resin (four from Fossellone and six from Sant’Agostino) are typologically and technologically variable. They include side and transverse scrapers, one Levallois flake and one unretouched flake. Most pieces have a length between 2.4–3.2 cm; the unretouched flake is 6 cm long but the indication of hafting is uncertain. Nevertheless it seems that hafting was applied to many pieces, regardless of their type, size and technological provenience. The use of resin as hafting material also suggests common fire use, as documented by evidence of burnt lithics at both sites: 12.6% at Sant’Agostino layer A1 (202 of 1608 retouched and debitage pieces) and 9.2% at Fossellone layer 23 alpha (43 of 467 retouched and debitage pieces). At Fossellone layer 23 charcoal and one fireplace are also documented [52]. However, as indicated above, none of the tools analyzed in this paper show thermal scars or other evidence of heating so the presence of resin cannot be considered as an accident due to exposure to burning resinous wood. Other forms of post-excavation contamination are excluded since the stone artifacts were put in plastic bag and were stored in drawers in the Pigorini Museum, the Archaeology Department in Pisa and the Italian Institute of Human Paleontology in Anagni.

Based on experimental butchering, it was suggested that the small Middle Paleolithic tools of Latium sites could be used by hand and that hafting did not improve their functionality [48]. Our evidence proves that hafting was used by Neandertals on the small tools of Latium during MIS 3. In Europe the spatial and geographic evidence of hafting and use of plant-based adhesives, strongly suggests that between MIS 7 and MIS 3 hafting was a stable component of Neandertal technological behavior.

## Supporting information

S1 File Sites and assemblages: Text and figures. (PDF)

S2 File GC/MS procedures, instrumentation and figures. (PDF)
