# Correction: Hafting of Middle Paleolithic tools in Latium (central Italy): New data from Fossellone and Sant’Agostino caves

**DOI:** 10.1371/journal.pone.0223714

**Published:** 2019-10-03

**Authors:** The PLOS ONE Staff

## Notice of Republication

An incorrect version of Fig 1 was published in error. Additionally, a Supporting Information file was incorrectly included in the originally published article. The publisher apologizes for these errors. This article was republished on September 27, 2019 to correct for these errors. Please download this article again to view the correct version.
