# Supplementary material for: Hafting of Middle Paleolithic tools in Latium (central Italy): New data from Fossellone and Sant’Agostino caves
Source: PLoS One. 2019 Jun 20;14(6):e0213473. doi: 10.1371/journal.pone.0213473 (PMC6586293; doi:10.1371/journal.pone.0213473)
Supplement: S1 File — (PDF) [file pone.0213473.s001.pdf]

Supporting Information

Hafting of Middle Paleolithic tools from Latium (central Italy): New data from Fossellone and Sant'Agostino caves

Ilaria Degano, Sylvain Soriano, Paola Villa*, Luca Pollarolo, Jeannette J. Lucejko, Zenobia Jacobs, Katerina Douka, Silvana Vitagliano, Carlo Tozzi

*** To whom correspondence should be addressed. E-mail: villap@colorado.edu**

S1 File. Sites and assemblages: text and figures.

This PDF file contains:

- Grotta del Fossellone. Stepped section opened in 2012 and location of 2013 OSL samples (Figure A).
- The Early Aurignacian lithic industry from Fossellone, layer 21: carinated endscrapers and bladelet production (Figures B-D).
- Sample of Pontinian pebbles (Figure E)
- The Middle Paleolithic industry from Fossellone layer 23 alpha (Figures F-G)
- The classical bipolar flaking and the Pontinian variant (Figure H)
- Grotta di Sant'Agostino. View of the inside. Limits of excavation trenches are not visible (Figure I).
- The Middle Paleolithic industry from Sant'Agostino cave (Figure J)
- References

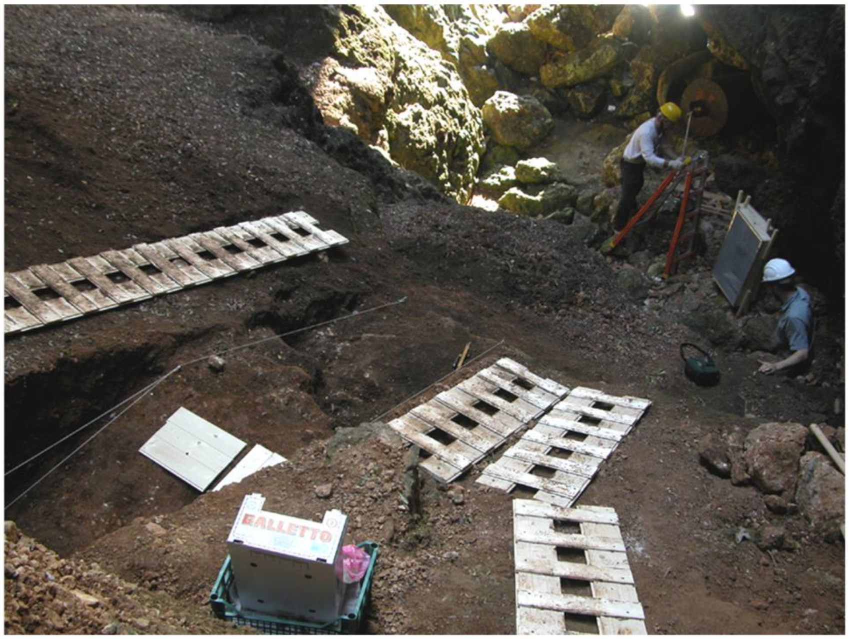

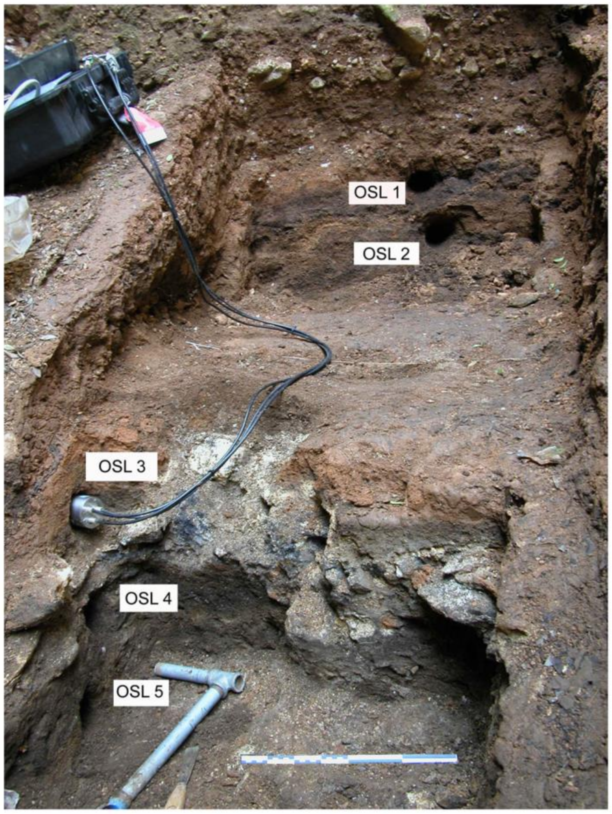

Figure A. Grotta del Fossellone. Ongoing excavation (top) and stepped section with location of OSL samples (bottom).

The Early Aurignacian lithic industry from Fossellone (layer 21): carinated endscrapers and bladelet production

A sample of 1263 lithic pieces from Fossellone layer 21 housed at the Museo Pigorini was examined and classified technologically. This sample, mainly composed of byproducts, is undoubtedly biased. Typologically diagnostic pieces, i.e. carinated endscrapers and Aurignacian retouched blades, are rare or absent whereas they are described as abundant by previous scholars; moreover the smallest bladelets are almost completely lacking. The scarcity of the first kind of pieces is likely due to sorting done by previous scholars and curation mistakes while the second is undoubtedly a recovery bias due to the sieving mesh used by Blanc when excavating the site. These biases did not prevent the reconstruction of the chaîne opératoire because our sample was completed by a small number of carinated endscrapers from the Italian Institute of Human Paleontology and Prehistory at Anagni to provide additional data.

Aurignacian flint knappers from Fossellone used the bipolar technique to produce thick blanks to be transformed into carinated endscrapers. This technique was designed to create suitable platform on ovoid flint pebbles, but it has frequently failed. Numerous waste products were discarded after unsuccessful hammering, that is pebble with too short removal negatives (Fig B: A and Fig C: A) or with irregular negatives (Figs B: B and C: B), longitudinal fragment of pebble with triangular cross-section (Fig B: D and Fig C: C), too thin bipolar flakes (Fig B: E) or pebbles with scaled negatives of removals on both faces (Figure B: C). Suitable blanks were first shaped by removing a series of cortical and partly cortical flakes on both sides up to one of the extremities. Unsuccessful or discarded roughouts are examples of this phase (Fig C: E-G). This shaping is close to the one described by Chiotti and Cretin (2011) for the roughouts of carinated scrapers from the Early Aurignacian from Castanet rockshelter. The first bladelet might retain cortical remnants together with negatives of shaping flakes converging from each flank of the endscraper (Fig C: D). Flakes removed at the junction between the front and the sides of the carinated endscrapers for the maintenance of convexities are bearing the distal part of the negatives of previous targeted bladelets (Fig C: H, O, P) but they have smaller platform (they are less "notching" the edge of the platform) than those described for such type of bladelet cores (Aubry et al., 1995; Chiotti, 2000; Tixier and Inizan, 1981). Broad bladelets (Fig C: I), with negatives of narrow and tiny ones on dorsal (Fig C: K-N), were involved in management of front convexities. The fronts of carinated endscrapers are not as large as usually described for Early Aurignacian (Le Brun-Ricalens, 2005b; Pelegrin and O'Farrell, 2005) but it might be explained by the limited size of blanks. On these fronts there are removals corresponding to both byproducts and endproducts (Fig D). Bladelets fitting with the smallest negatives of removals on carinated endscrapers, those from targeted products that could be less than 20 mm in length and 3 to 5 mm in width, are almost lacking in our sample due to sampling bias.

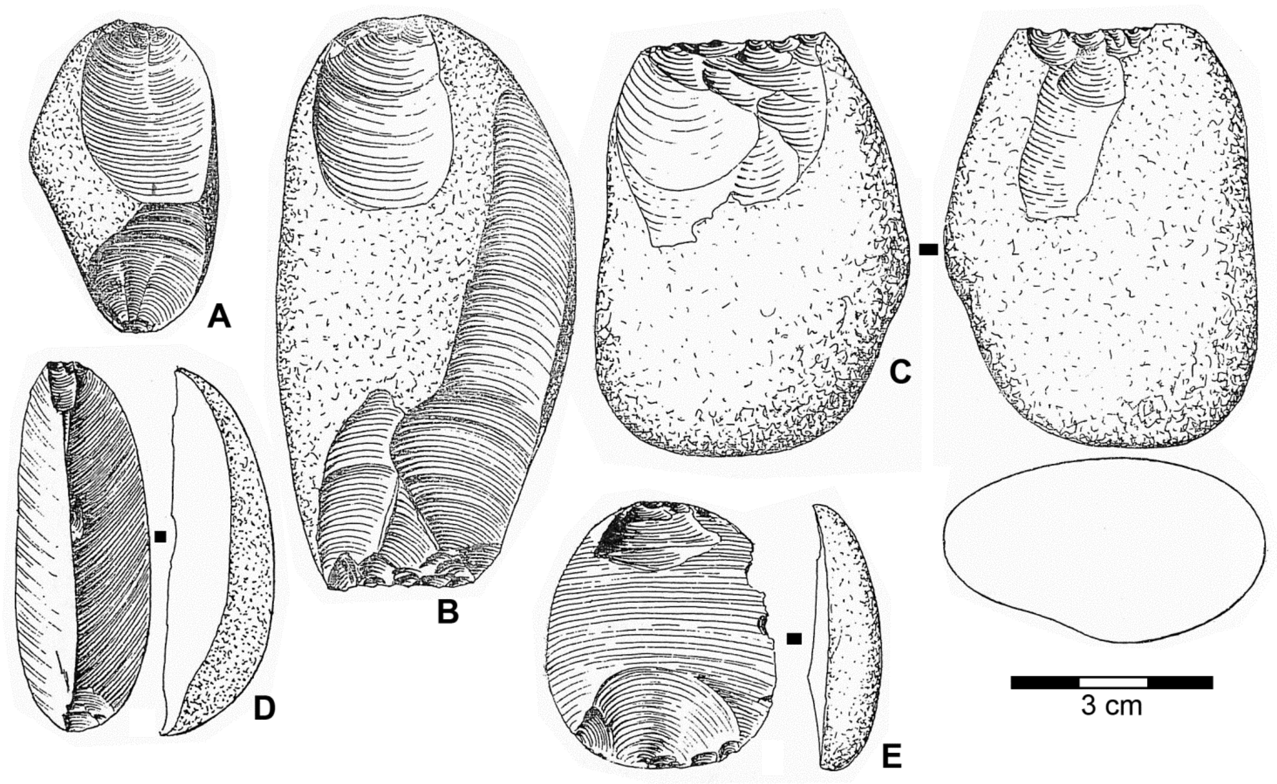

Figure B. Fossellone Cave. Stone artifacts from layer 21. (A-E) Waste products discarded after unsuccessful bipolar hammering. Modified from (Blanc and Segre 1953). Courtesy of the Istituto Italiano di Paleontologia Umana.

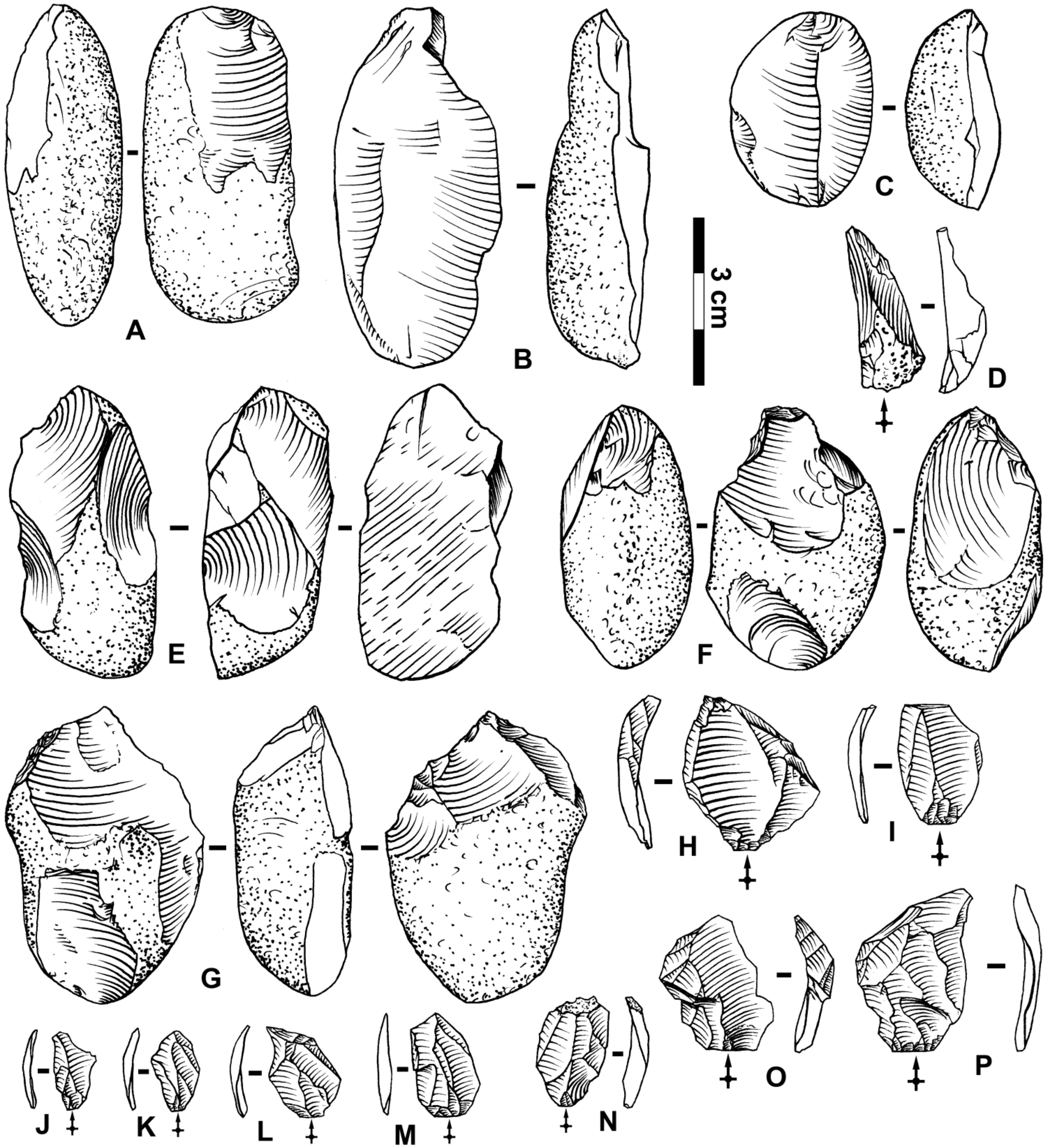

Figure C. Fossellone Cave. Stone artifacts from layer 21. (A-C) Waste products discarded after unsuccessful bipolar hammering; (D) first bladelet extracted on a carinated endscraper; (E-G) roughouts of carinated endscrapers; (H, I, O, P) maintenance flakes removed from the flanks of carinated endscrapers; (J) bladelet; (K-N) broad bladelets managing front convexities on a carinated endscraper. Drawings S. Soriano.

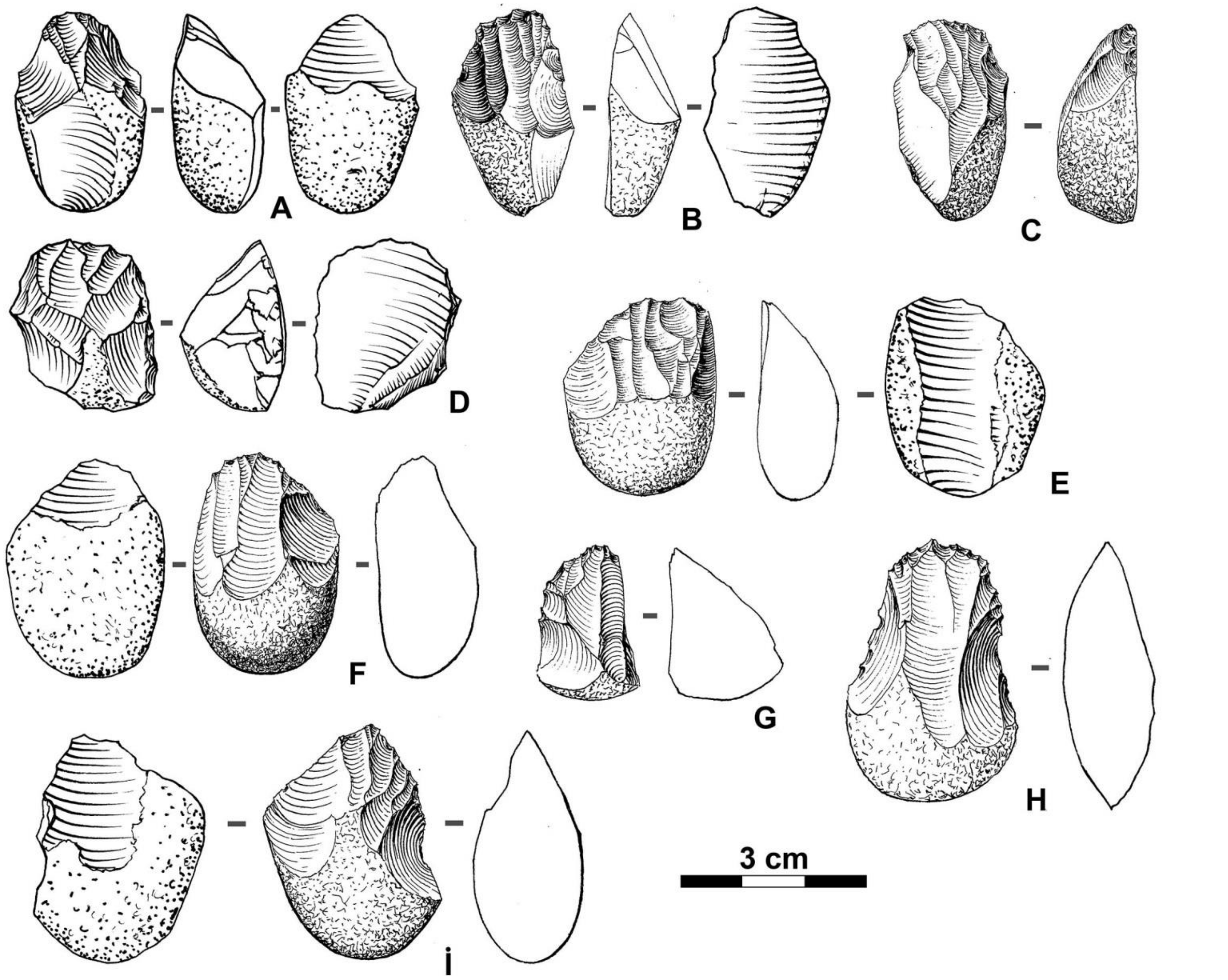

Figure D. Fossellone Cave. Stone artifacts from layer 21. (A, D) Carinated endscrapers. Drawings S. Soriano (A, D) and modified from unpublished drawings from A.C. Blanc project (B, C, E - I). Courtesy of the Istituto Italiano di Paleontologia Umana.

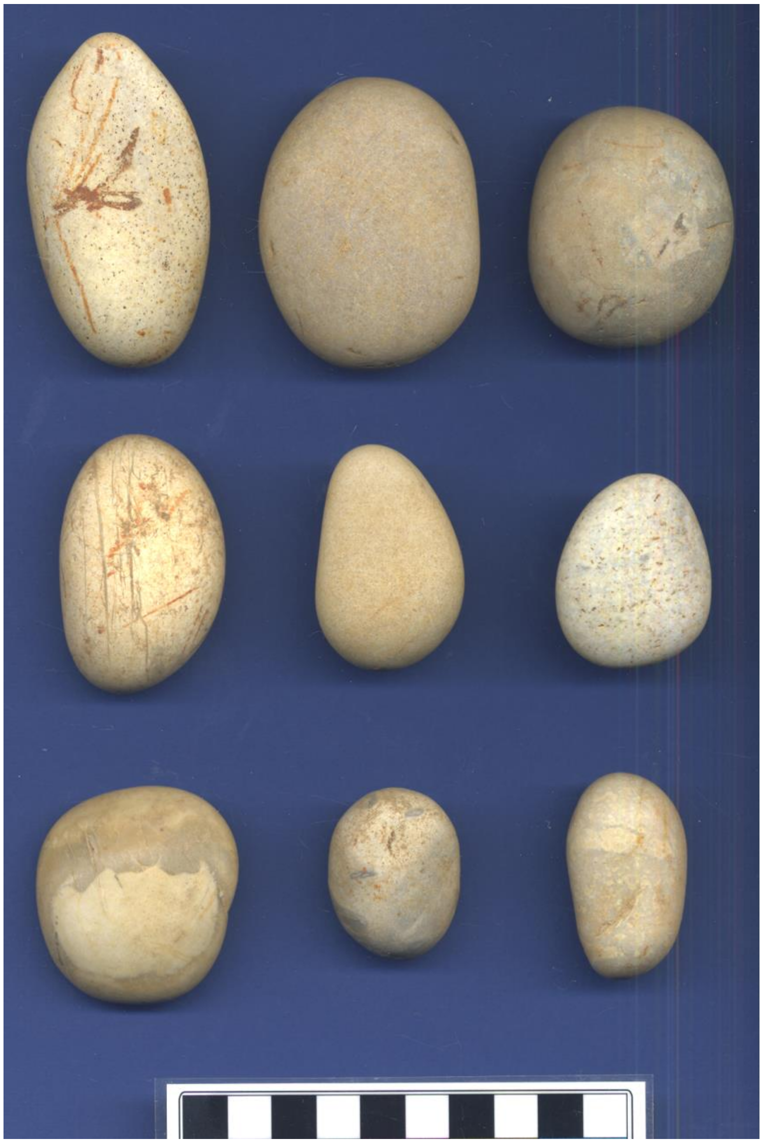

Figure E. Sample of Pontinian pebbles.

The Middle Paleolithic industry from Fossellone layer 23 alpha

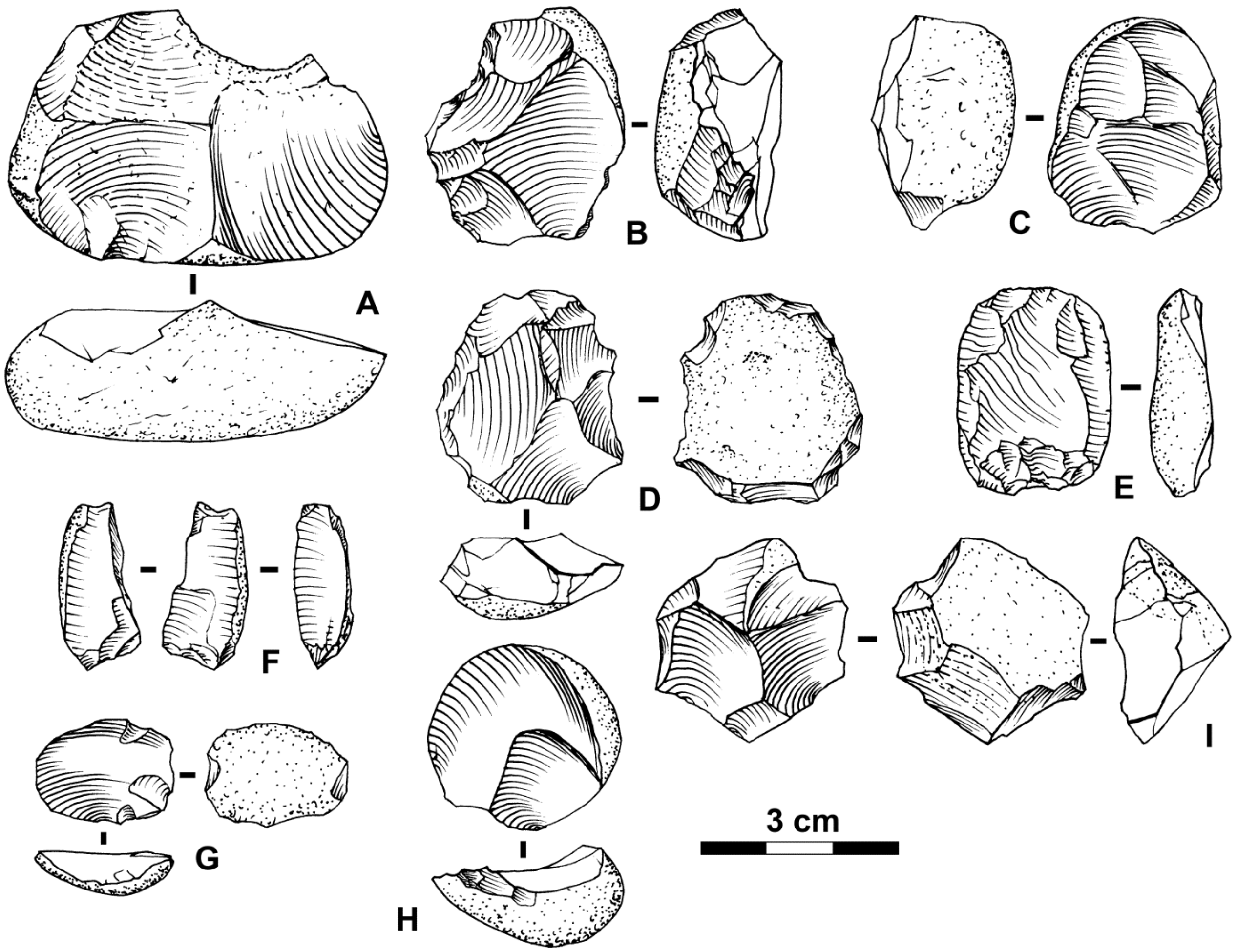

Figure F. Fossellone Cave. Stone artifacts from layer 23 alpha. Centripetal cores (A, H); Discoidal cores (B, I); Unipolar core (C); Levallois core (D); Bipolar cores (E, F); Core with a single overwhelming removal (G). Drawings S. Soriano.

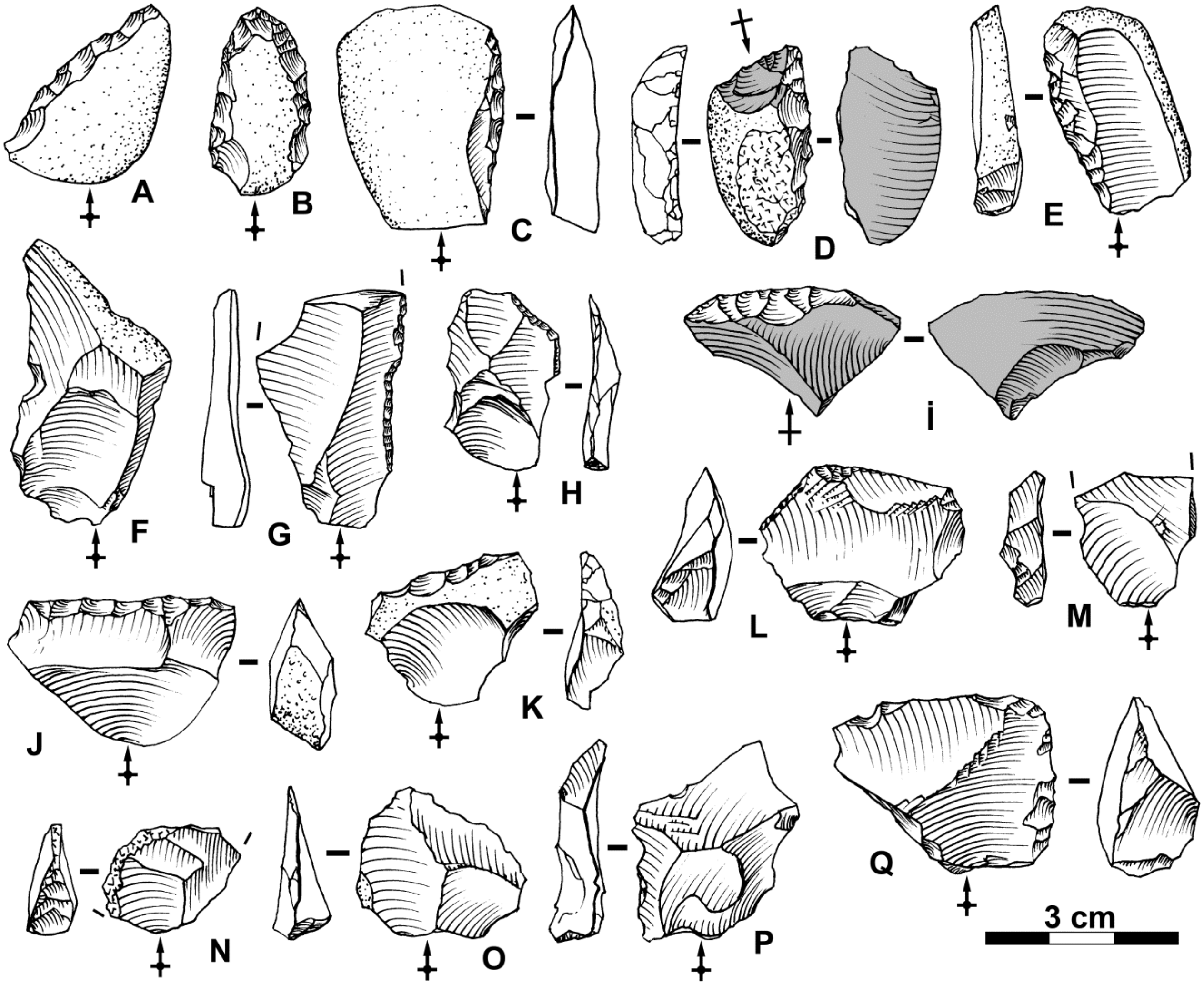

Figure G. Fossellone Cave. Stone artifacts from layer 23 alpha. (A-C) Scrapers on cortical flakes; (D) *Déjeté* scraper on recycled cortical flake; (E-G) Scrapers on flakes from with scars of successive series of unidirectional parallel removals; (H) Levallois flake; (I) Transverse scraper on recycled flake; (J, K) Scrapers on centripetal flakes, possibly from discoidal production; (L) Centripetal flake, possibly from discoidal production; (M-Q) Chordal flakes and pseudo-Levallois points from discoidal production. Surfaces shaded in grey correspond to double patina (older pieces retouched after patina developed). Drawings S. Soriano.

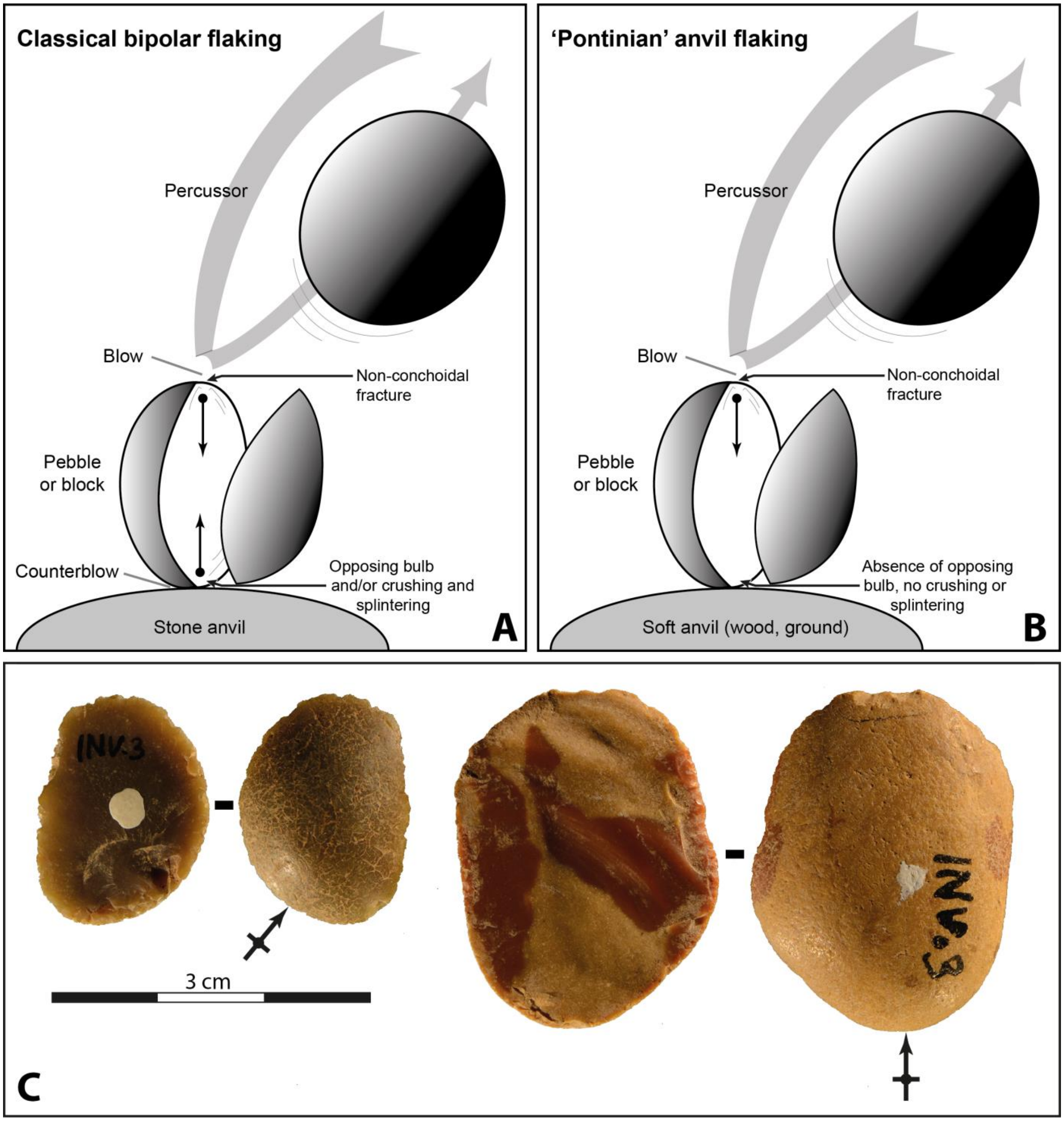

Figure H. Differences between the classical bipolar flaking and the 'Pontinian" anvil flaking. (A) With the classical bipolar flaking the counterblow results in the formation of an opposing bulb and/or crushing and splintering. (B) With a soft anvil the counterblow does not generate any features on flakes or cores. (C) Two flat flakes by the "Pontinian" anvil flaking, from Sant'Agostino cave. After Soriano and Villa, 2017, fig. 16.

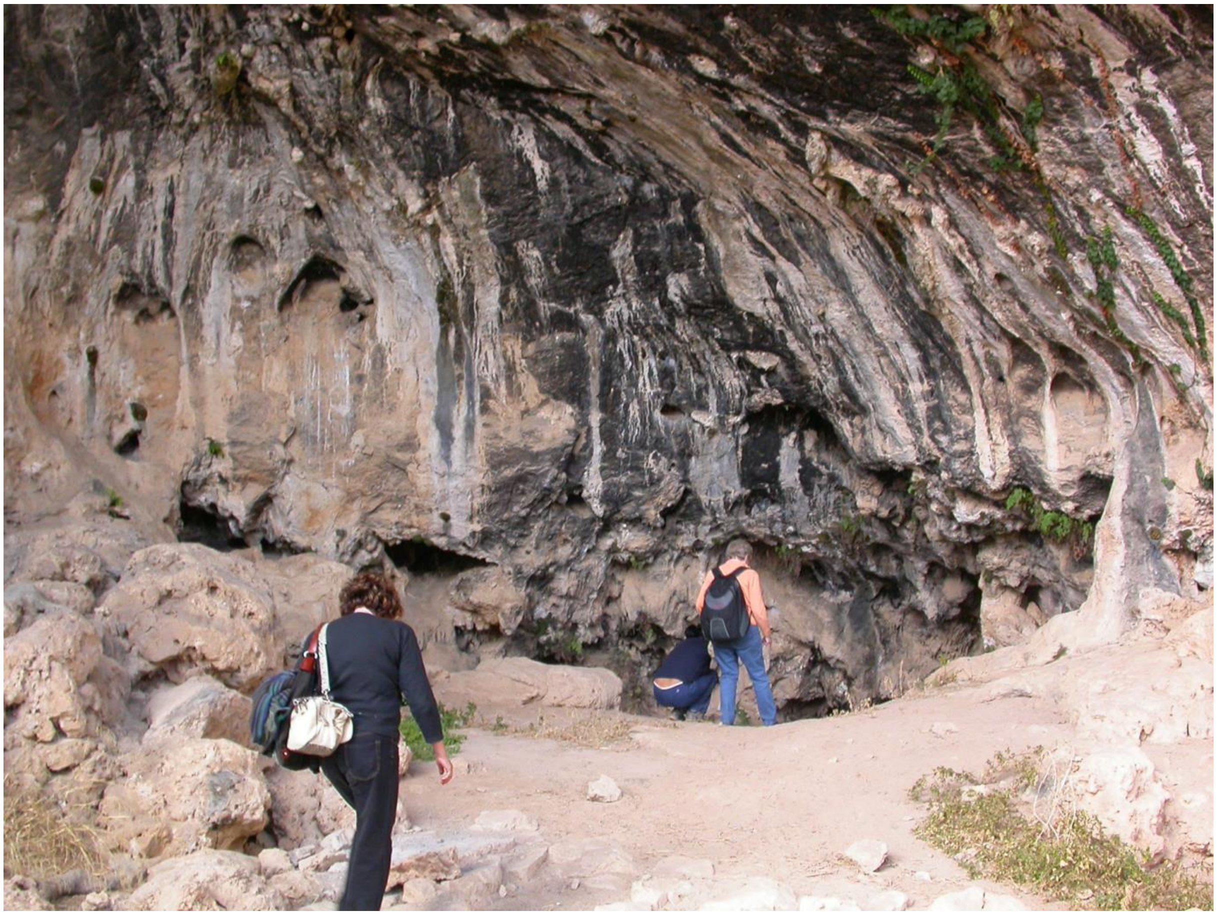

Figure I. Grotta di Sant'Agostino. View of the inside. Limits of excavation trenches are not visible.

The Middle Paleolithic industry from Sant'Agostino cave

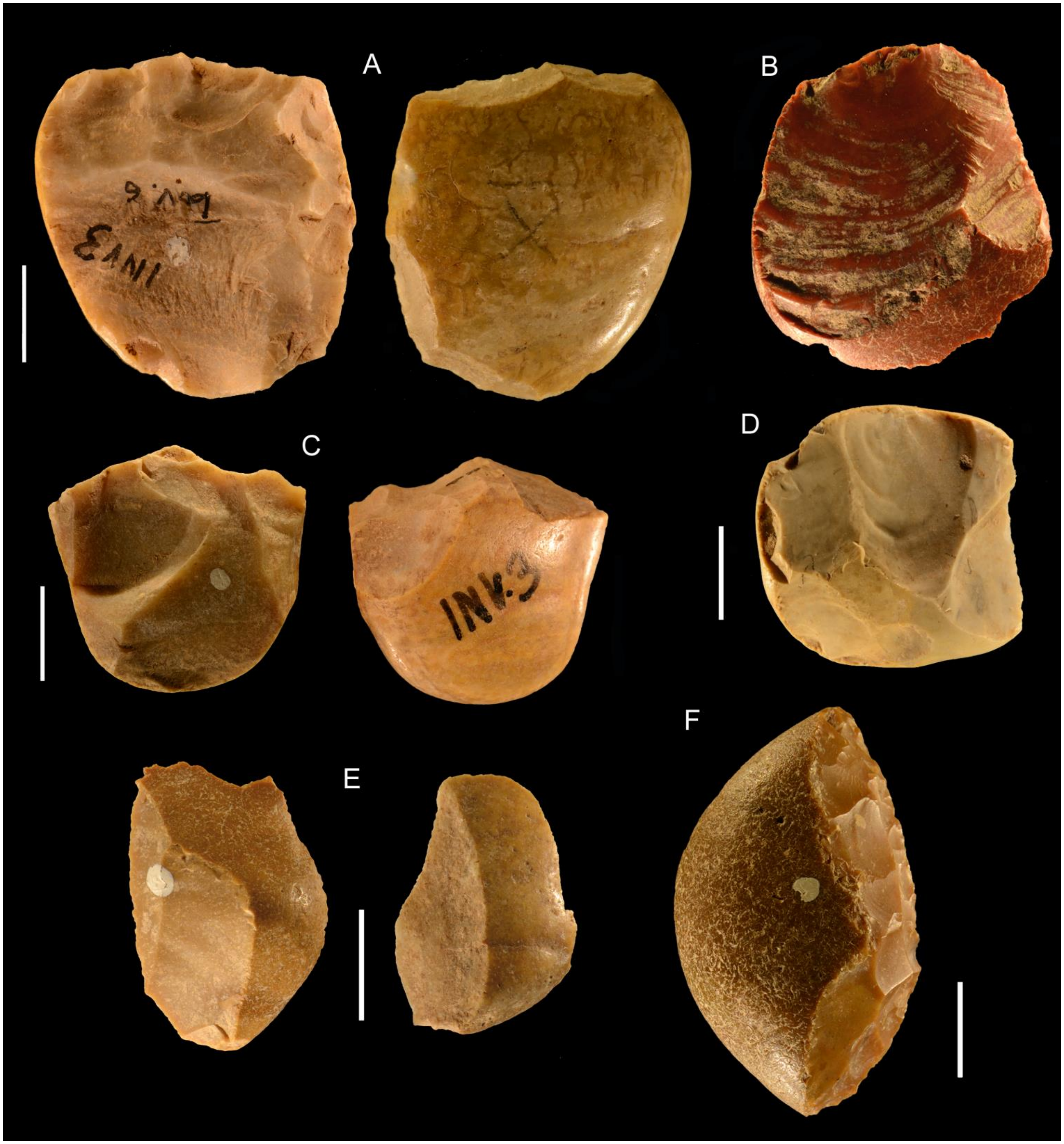

Figure J. Sant'Agostino layer A1. (A) Levallois core. (B) Core with a flat removal. (C) Core with a series of unidirectional parallel removals. (D) Core with multiple flat removals. (E) Two flakes with lateral and distal cortex. (F) Side scraper. Scale bar = 1 cm.

References

Aubry, T., Detrain, L., Kervazo, B., 1995. Les niveaux intermédiaires entre le Gravettien et la Solutréen de l'Abri Casserole (Les Eyzies de Tayac). Bulletin de la Société Préhistorique Française 92, 296–301.

Blanc, A.C., Segre, A.G., 1953. La Grotta del Fossellone (Grotte du Grand Fossé), in: Blanc, A.C., Segre, A.G. (Eds.), Excursion Au Mont Circé. Le Volcan Latial – Le Mont Circé. INQUA, IVe Congrès International, Rome, Pise, 1953. INQUA, Rome, pp. 37–85.

Chiotti, L., 2000. Lamelles Dufour et grattoirs aurignaciens (carénés et à museau) de la couche 8 de l'abri Pataud, Les Eyzies-de-Tayac, Dordogne. L'Anthropologie 104, 239–263.

Chiotti, L., Cretin, C., 2011. Les mises en forme de grattoirs carénés / nucléus de l'aurignacien ancien de l'abri Castanet (Sergeac, Dordogne). PALEO 22. 69–84.

Le Brun-Ricalens, F., 2005. Reconnaissance d'un "concept technoculturel" de l'Aurignacien ancien ? Modalités, unités et variabilités des productions lamellaires du site d'Hui (Beauville, Lot-et-Garonne, France) : significations et implications, in: Le Brun-Ricalens, F. (Ed.), Productions Lamellaires Attribuées à l'Aurignacien : Chaînes Opératoires et Perspectives Technoculturelles, ArchéoLogiques. Presented at the XIVe congrès de l'UISPP, Liège, 2-8 septembre 2001, Musée national d'Histoire et d'Art, Luxembourg, pp. 157–190.

Pelegrin, J., O'Farrell, M., 2005. Les lamelles retouchées ou utilisées de Castanet, in: Le Brun-Ricalens, F. (Ed.), Productions Lamellaires Attribuées à l'Aurignacien : Chaînes Opératoires et Perspectives Technoculturelles, ArchéoLogiques. Presented at the XIVe congrès de l'UISPP, Liège, 2-8 septembre 2001, Musée national d'Histoire et d'Art, Luxembourg, pp. 103–121.

Soriano, S., Villa, P., 2017. Early Levallois and the beginning of the Middle Paleolithic in central Italy. PLoS One 12, e0186082. https://doi.org/10.1371/journal.pone.0186082

Tixier, J., Inizan, M.-L., 1981. Ksar'Akil. Stratigraphie et ensembles lithiques dans le Paléolithique supérieur. Fouilles 1971 à 1975, in: Préhistoire Du Levant. Chronologie et Organisation de l'espace Depuis Les Origines Jusqu'au VIe Millénaire, Colloques Internationaux Du CNRS, N°598. Éditions du CNRS, Paris, pp. 353–368.
