# Supplementary material for: Hafting of Middle Paleolithic tools in Latium (central Italy): New data from Fossellone and Sant’Agostino caves
Source: PLoS One. 2019 Jun 20;14(6):e0213473. doi: 10.1371/journal.pone.0213473 (PMC6586293; doi:10.1371/journal.pone.0213473)
Supplement: S2 File — (PDF) [file pone.0213473.s002.pdf]

Supporting Information

Hafting of Middle Paleolithic tools from Latium (central Italy): New data from Fossellone and Sant'Agostino caves

Ilaria Degano, Sylvain Soriano, Paola Villa*, Luca Pollarolo, Jeannette J. Lucejko, Zenobia Jacobs, Katerina Douka, Silvana Vitagliano, Carlo Tozzi

*** To whom correspondence should be addressed. E-mail: villap@colorado.edu**

S2 File. GC/MS procedures, instrumentation and figures.

This PDF file includes:

1. Chemical analysis by Gas Chromatography/Mass Spectrometry.

Apparatus
Sample treatment
Replicate analyses
Comparing the environmental sediment samples and the stone tool samples

2. Chromatograms of environmental blanks (Figures A-C)

3. Chromatograms of artifacts from Fossellone with significant results F3, F4 and F5 (Figures D-F)

4. Chromatograms of artifacts from Sant'Agostino with significant results AGO 2, AGO 3, AGO 5, AGO 6 and AGO 10 (Figures G, H, I, J, K).

5. Chromatograms of artifacts F2, F6, F10 from Fossellone cave (Figures L,M,N)

6. Chromatogram of artifact AGO1 from Sant'Agostino cave (Figure O)

7. Figure P. Fossellone cave. Stone artifacts from layer 23 alpha (F2, F6, F7) and 23 gamma (F10). These were sampled but none gave significant results.

8. References

Chemical analysis by Gas Chromatography/Mass Spectrometry

Apparatus

The GC/MS instrumentation consists in a 6890N Network GC System (Agilent Technologies, Palo Alto, CA, USA) equipped with a PTV injector and coupled to a 5973 MS detector with quadrupole analyzer. MS parameters: electron impact ionization (EI, 70 eV) in positive mode; ion source temperature 230°C; scan range 50-700 m/z; interface temperature 280°C.
GC separation was performed on a HP-5MS column (J&W Scientific, Agilent Technologies, stationary phase 5% phenyl–95% methylpolysiloxane, 30m length, 0.25 mm i.d., 0.25 µm film thickness) connected to a deactivated fused silica precolumn (J&W Scientific, Agilent Technologies, 2 m length, 0.32 mm i.d.).
<u>GC conditions for the lipid-resinous fraction</u>: the PTV injector was used in splitless mode at 300 °C and the chromatographic oven was programmed as follows 80° C, for 2 min isothermal, 10 °C/min up to 200 °C, 4 min isothermal, 6 °C/min up to 280°C, 40 minutes isothermal; constant He flow 1.2 ml/min, injector temperature 280 °C.
<u>GC conditions for the proteic fraction</u>: the PTV injector was used in splitless mode at 220 °C, and the chromatographic oven was programmed as follows: initial temperature 100 °C, isothermal for 2 min, then 4 °C/min up to 280 °C, 280 °C isothermal for 15 min.
<u>GC conditions for the saccharide fraction</u>: the PTV injector was used in splitless mode at 250 °C and the chromatographic oven was programmed as follows: 50 °C isothermal for 2 min, 5 °C/min up to 190 °C, 190 °C isothermal for 20 min 5 °C/min up to 280 °C, 280 °C isothermal for 15 min.

Sample treatment

The already published combined analytical procedure employed, allows identification of polysaccharide, proteinaceous, glycerolipid materials, as well as waxes and terpenoid resins in the same micro-sample (Andreotti et al. 2010; Lluveras et al., 2010). The procedure is based on a multistep chemical pre-treatment of the sample, to obtain three different fractions to be analyzed separately by GC/MS: an amino acidic, a saccharide and a lipid-resinous fraction. First proteins and polysaccharide materials were subjected to ammonia extraction in order to separate them from lipid and resinous materials. Proteins and sugars were separated afterwards by monolithic sorbent tip technology with a C4 stationary phase and purified before hydrolysis. Three fractions were generated and analyzed separately by GC/MS. Lipids and resins were subjected to saponification assisted by microwaves and the three fractions were separately derivatized.
The quantitative determination of amino acids, aldoses and uronic acids, aliphatic mono- and dicarboxylic acids is performed by using standard solutions, building calibration curves, and evaluating daily recoveries. Running blanks of the procedure highlighted a low level of contamination. The detection limit (LOD) and the quantitation limit (LOQ) of amino acids, aldoses, uronic acids, and fatty

and dicarboxylic acids were calculated. At a statistical significance level of 0.05, the LODs and LOQs obtained of the proteinaceous, glycerolipids and saccharide materials were as follows:
Proteinaceous material: LOD 0.14 μg; LOQ 0.27 μg;
Glycerolipids: LOD 0.35μg; LOQ 0.50μg;
Saccharide material: LOD 0.21 μg; LOQ 0.48 μg.
Proteinaceous materials were identified based on the percentage composition of 11 determined amino acids. Using these data as variables, multivariate statistical analysis, namely principal component analysis (PCA), was applied (XLSTAT 6.0, Addinsoft, France) on the correlation matrix of the data and the first two components account for 96.3% of the data. In the resulting score plots samples were represented together with the data set of 121 reference samples of animal glue, egg, and casein. The presence and absence of the sugars was used for the identification of the saccharide content by means of a decisional scheme already published in the literature.
The analysis of amino acids as well as the sugar and uronic acids did not yield significant results: the amount of analytes was below the detection limit of the procedure.

Replicate analyses

We replicated some of the analyses with different aliquots of the sample extracts, to obtain the best response possible for all the components of each sample. We do not systematically run duplicates, since this type of analysis is based on the detection of specific molecular markers and not on absolute concentration values. This is the reason why no quantitative parameters for the method were reported here. Nonetheless, our method has been validated and the results published in the cited reference and thus shall not be repeated here. Our laboratory is running blank analyses with the evaluation of environmental and procedure blanks for all the analytes for which an analytical standard is available (Andreotti et al., Anal. Chem., 2006, 78 (13), pp 4490–4500). In the case of archaeological residues, the comparison with the environmental (soil) samples is even more important than the comparison with the procedure blank, as presented in our paper.

Comparing the environmental sediment samples and the samples from the stone tools.

The comparison was based on the relative intensities of the chromatographic peaks with respect to the internal standard (see section 'Environmental blanks" in the main text). Our internal standard was tridecanoic acid, which is expected to be absent (or present in very minor abundances) in the actual samples. Although the response in terms of peak area of different organic species can be quite different, and the chosen internal standard cannot be considered a proxy for all the detected compounds in the chromatogram, the relative evaluation of soil contamination is currently used in the literature in this kind of studies (Garnier et al. 2002; Andreotti et al. 2006).

Chromatograms of environmental blanks (Figures A-C)

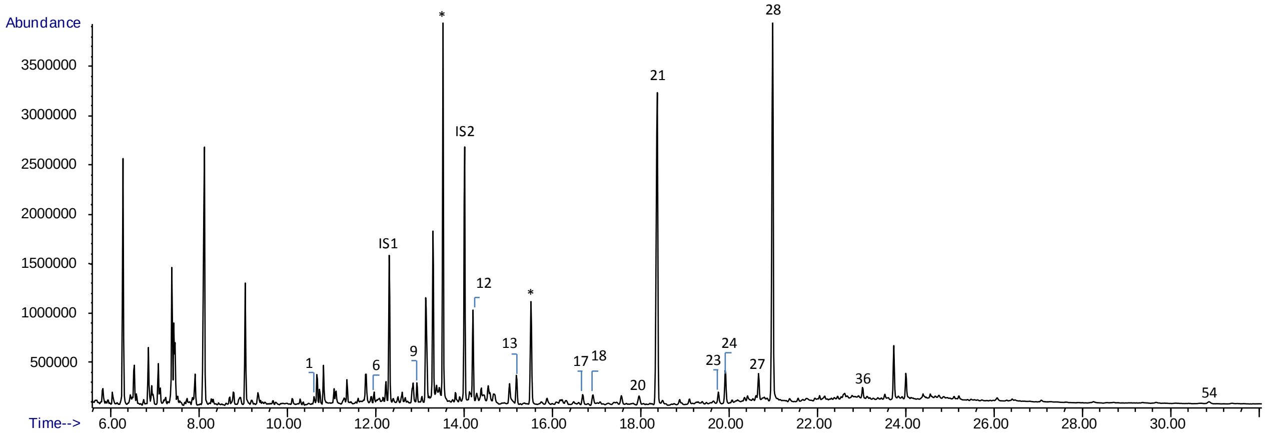

Figure A. Chromatographic profile obtained for lipid-resinous fraction of sample F8 (sediment from Fossellone). The labels refer to Table 8. IS1 = hexadecane, IS2 = tridecanoic acid, *: phthalate contamination.

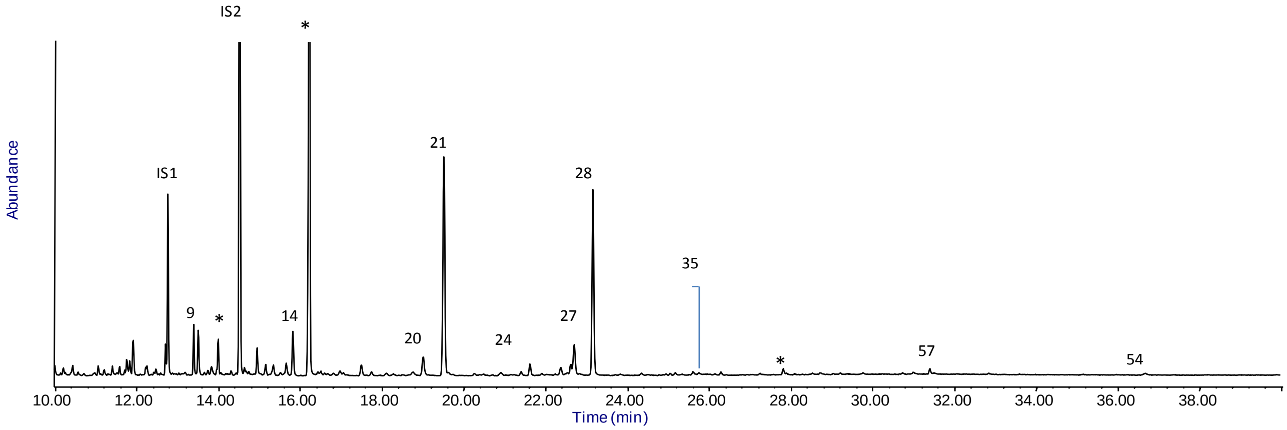

Figure B. Chromatographic profile obtained for lipid-resinous fraction of sample B1 (sediment on radius-ulna of *Cervus elaphus* from Sant'Agostino). The labels refer to Table 8. IS1 = hexadecane, IS2 = tridecanoic acid, *: phthalate contamination.

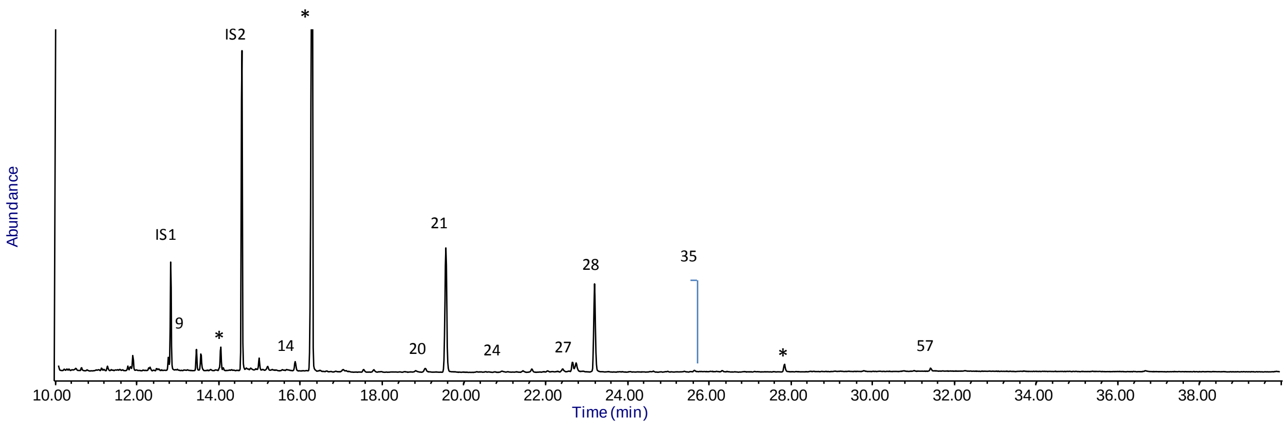

Figure C. Chromatographic profile obtained for lipid-resinous fraction of sample B2 (sediment on a *Bos* calcaneum from Sant'Agostino). The labels refer to Table 8. IS1 = hexadecane, IS2 = tridecanoic acid, *: phthalate contamination.

Chromatograms of artifacts from Fossellone with significant results F3, F4 and F5 (Figures D-F)

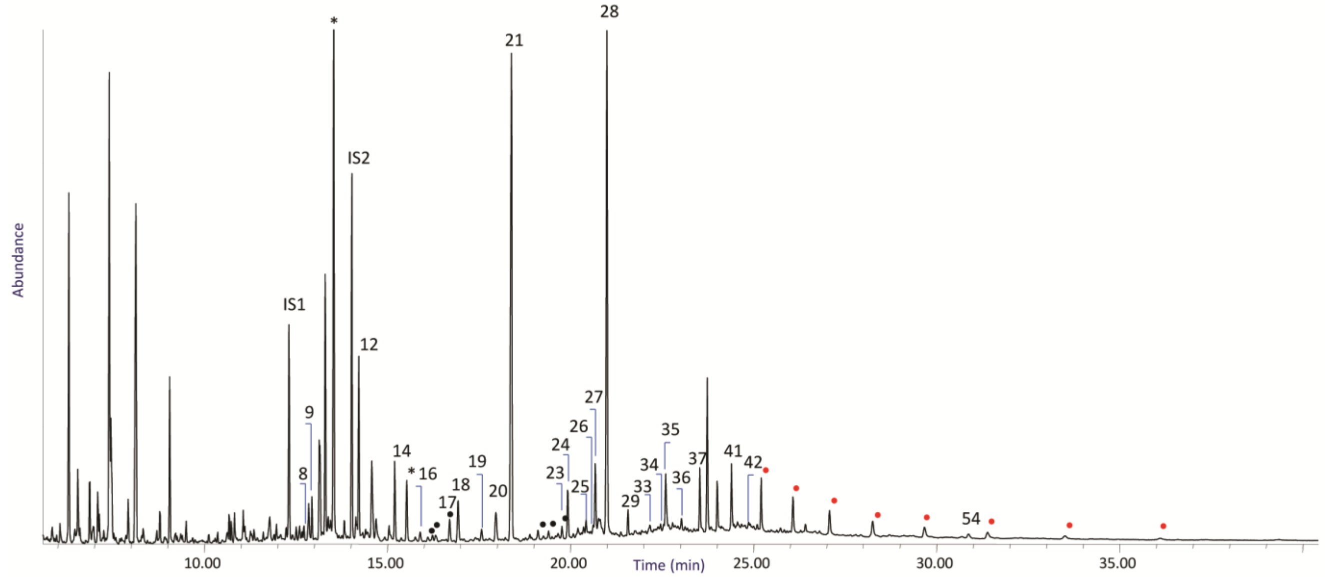

Figure D. Chromatographic profile obtained for lipid-resinous fraction of sample F3. The numbers refer to Table 8. IS1: hexadecane, IS2: tridecanoic acid, •: branched fatty acids, •: aliphatic hydrocarbons, *: phthalate contamination.

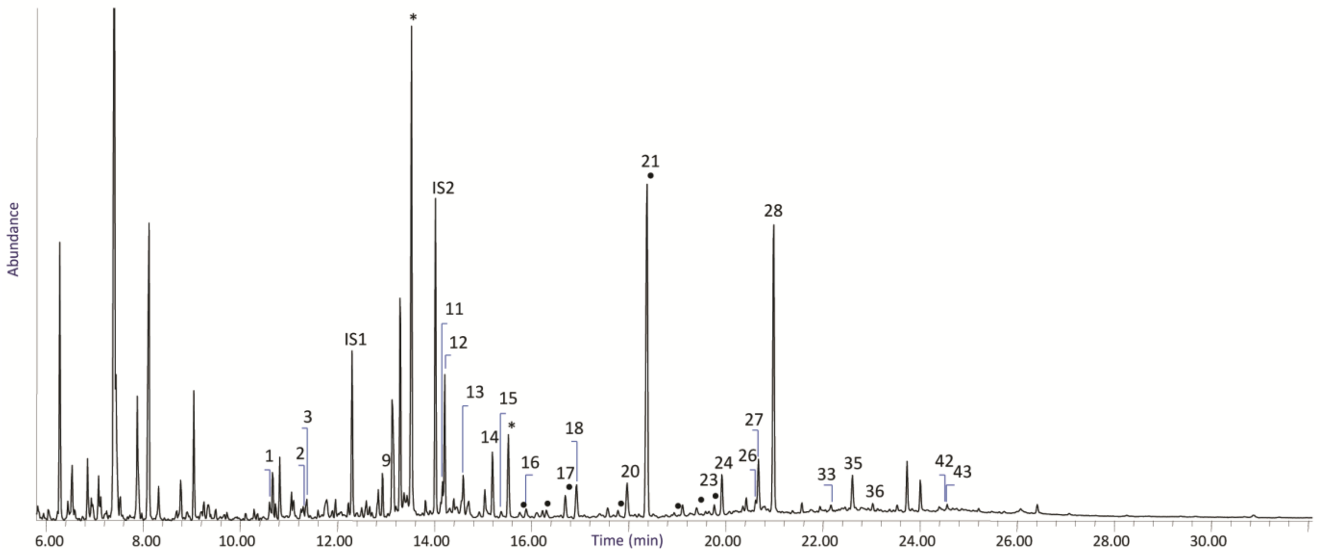

Figure E. Chromatographic profile obtained for lipid-resinous fraction of sample F4. The numbers refer to Table 8. IS1 = hexadecane, IS2 = tridecanoic acid, •: branched fatty acids, *: phthalate contamination.

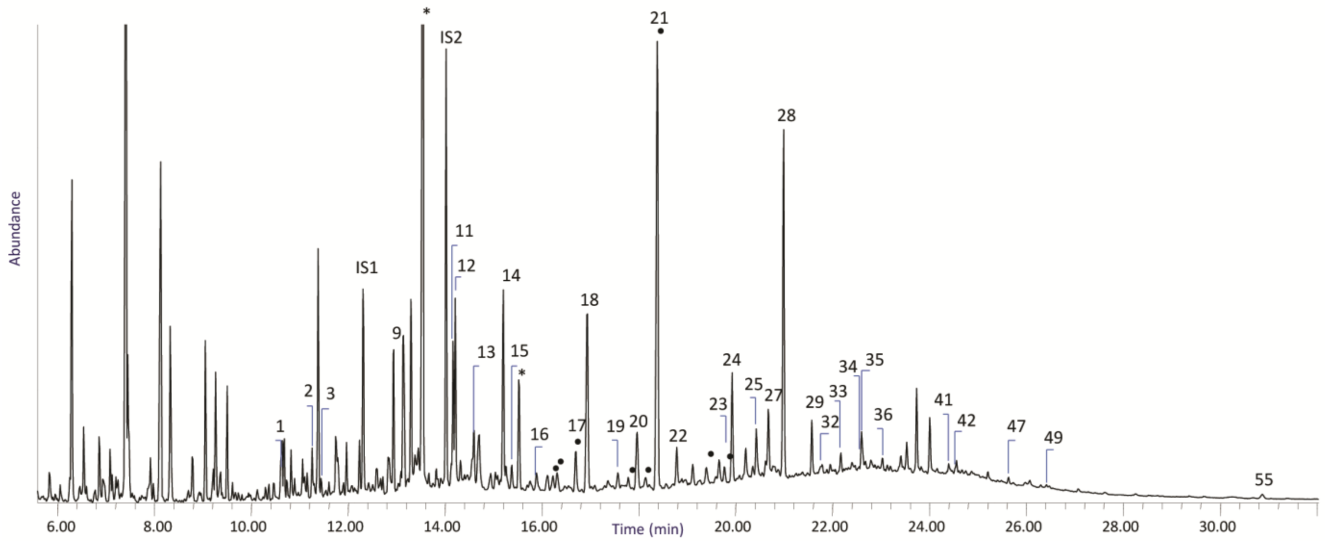

Figure F. Chromatographic profile obtained for lipid-resinous fraction of sample F5. The numbers refer to Table 8. IS1 = hexadecane, IS2 = tridecanoic acid, •: branched fatty acids, *: phthalate contamination.

Chromatograms of artifacts from Sant'Agostino with significant results AGO 2, AGO 3, AGO 5, AGO 6 and AGO 10 (Figures G- K).

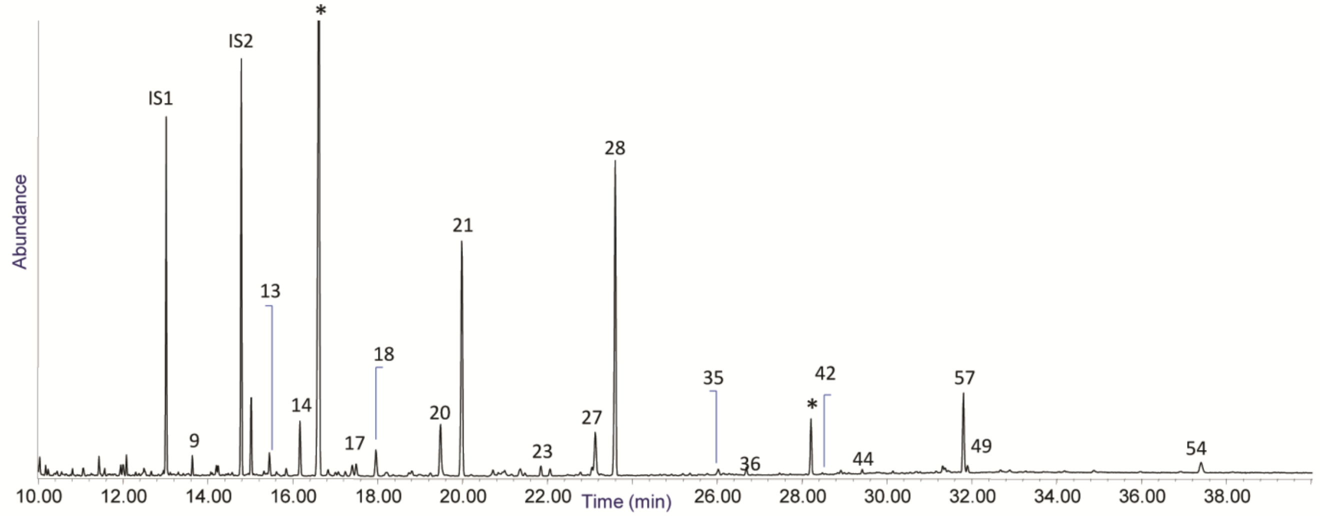

Figure G. Chromatographic profile obtained for lipid-resinous fraction of sample AGO 2, no. 114. The numbers refer to Table 8. IS1 = hexadecane, IS2 = tridecanoic acid, *: phthalate contamination

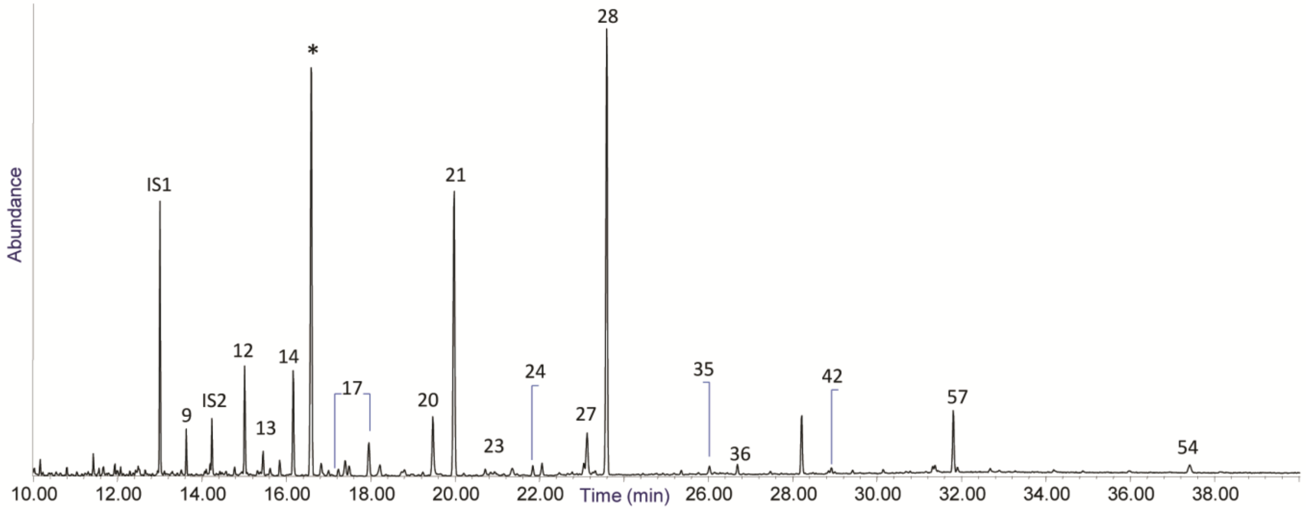

Figure H. Chromatographic profile obtained for lipid-resinous fraction of sample AGO 3 no. 211. The numbers refer to Table 8. IS1 = hexadecane, IS2 = tridecanoic acid, *: phthalate contamination.

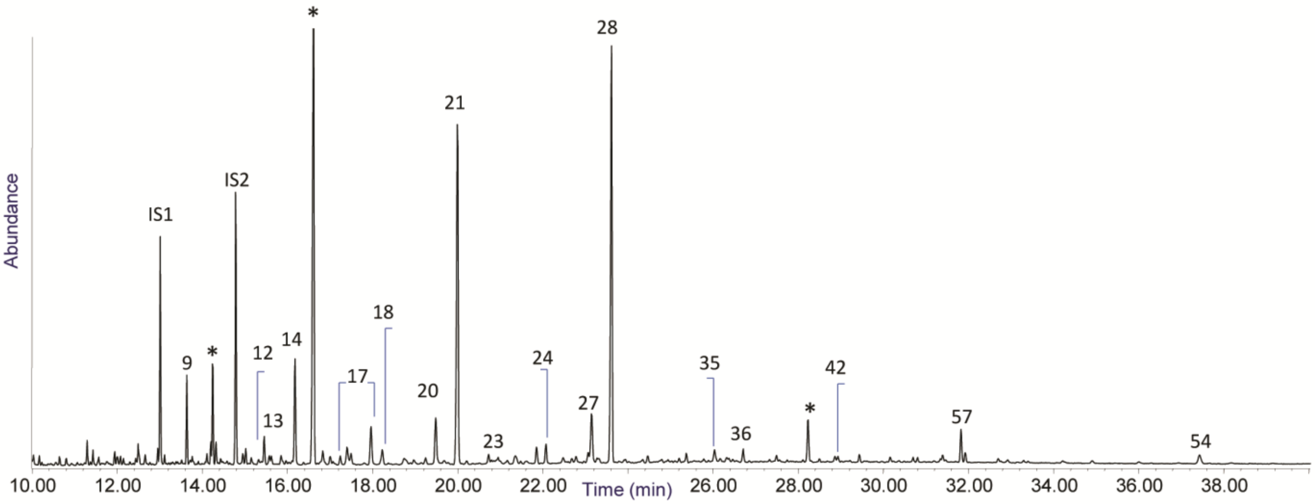

Figure I. Chromatographic profile obtained for lipid-resinous fraction of sample AGO5, no. 268. The numbers refer to Table 8. IS1 = hexadecane, IS2 = tridecanoic acid, *: phthalate contamination.

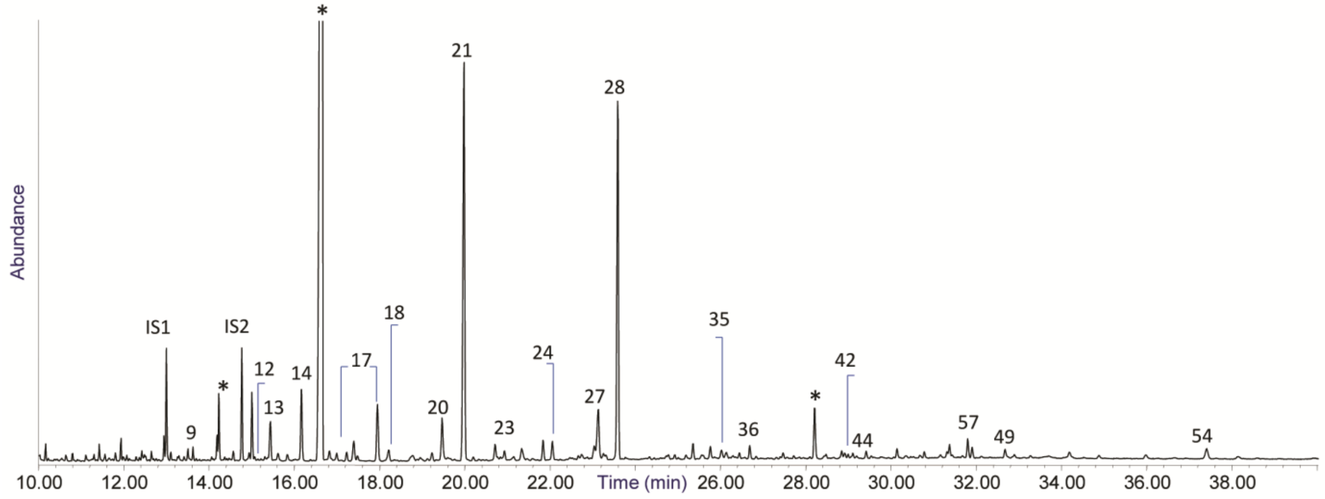

Figure J. Chromatographic profile obtained for lipid-resinous fraction of sample AGO 6, no. 362. The numbers refer to Table 8. IS1 = hexadecane, IS2 = tridecanoic acid, *: phthalate contamination.

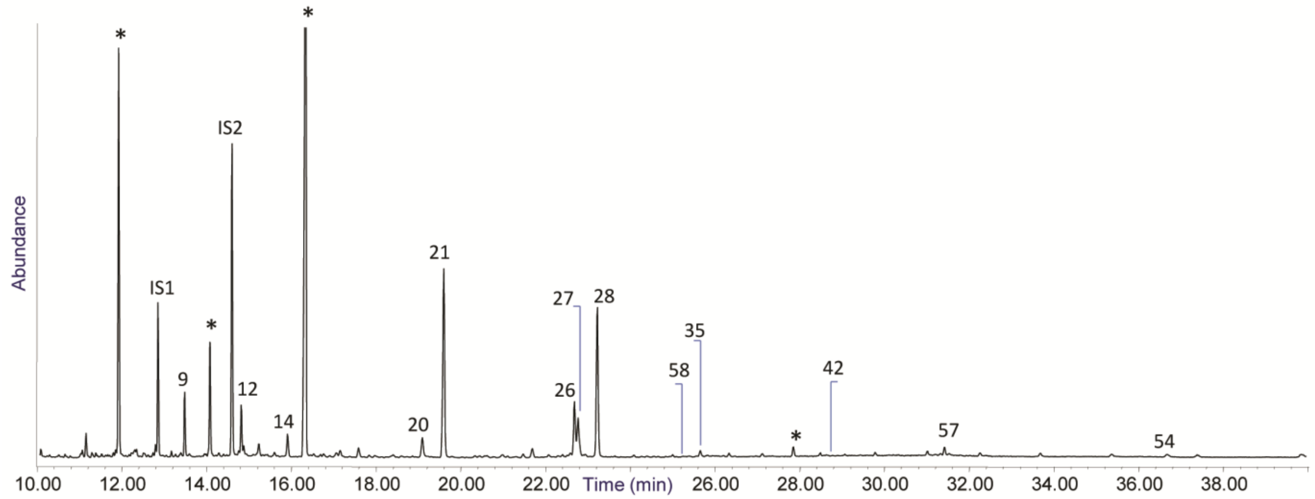

Figure K. Chromatographic profile obtained for lipid-resinous fraction of sample AGO 10, no. L2, Levallois flake. The numbers refer to Table 8. IS1 = hexadecane, IS2 = tridecanoic acid, *: phthalate contamination

Chromatograms of artifacts F2, F6, F10 from Fossellone cave (Figures L-N).

These pieces have not given significant results. The profile of Sample F7 is not illustrated here but it was equally unproductive.

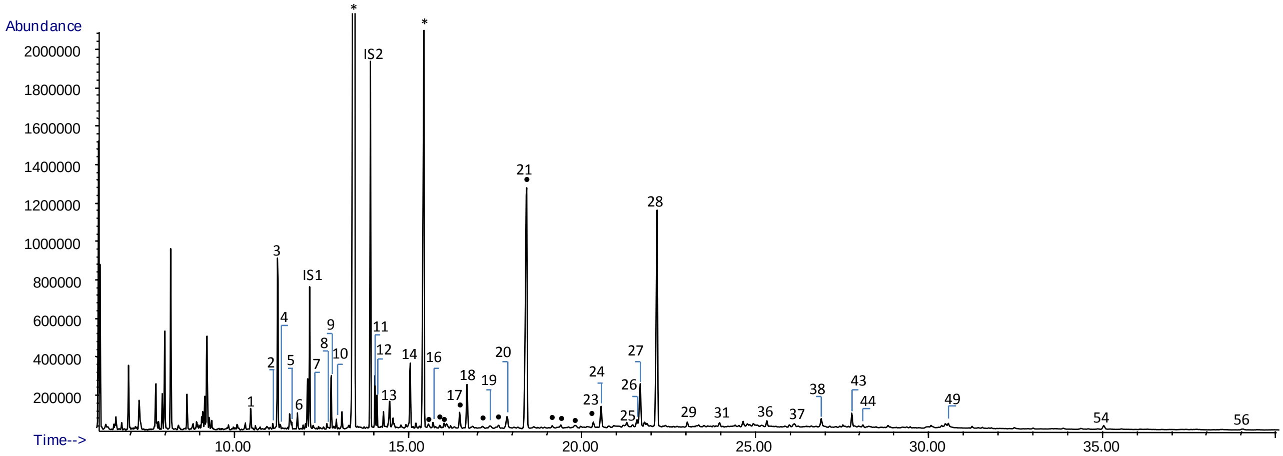

Figure L. Chromatographic profile obtained for lipid-resinous fraction of sample F2. The numbers refer to Table 8. IS1 = hexadecane, IS2 = tridecanoic acid, •: branched fatty acids, *: phthalate contamination.

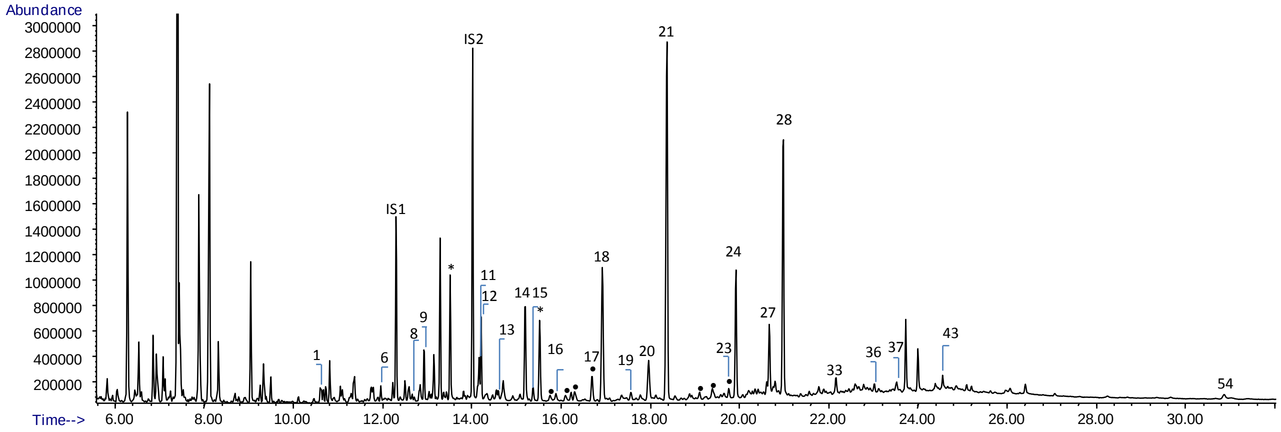

Figure M. Chromatographic profile obtained for lipid-resinous fraction of sample F6. The numbers refer to Table 8. IS1 = hexadecane, IS2 = tridecanoic acid, •: branched fatty acids, *: phthalate contamination.

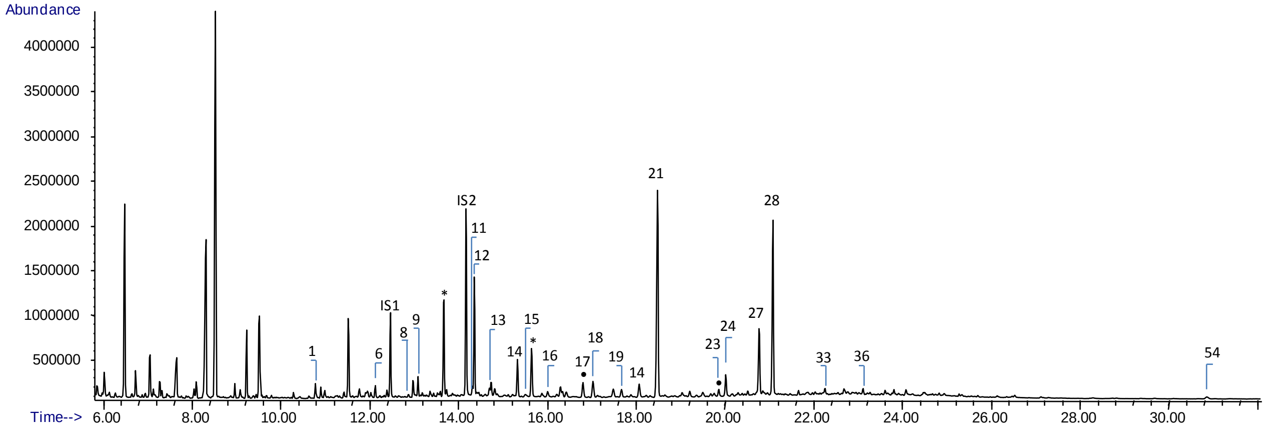

Figure N. Chromatographic profile obtained for lipid-resinous fraction of sample F10. The numbers refer to Table 8. IS1 = hexadecane, IS2 = tridecanoic acid, *: phthalate contamination.

Chromatogram of artifact AGO1 from Sant'Agostino cave

This piece has not given significant results.

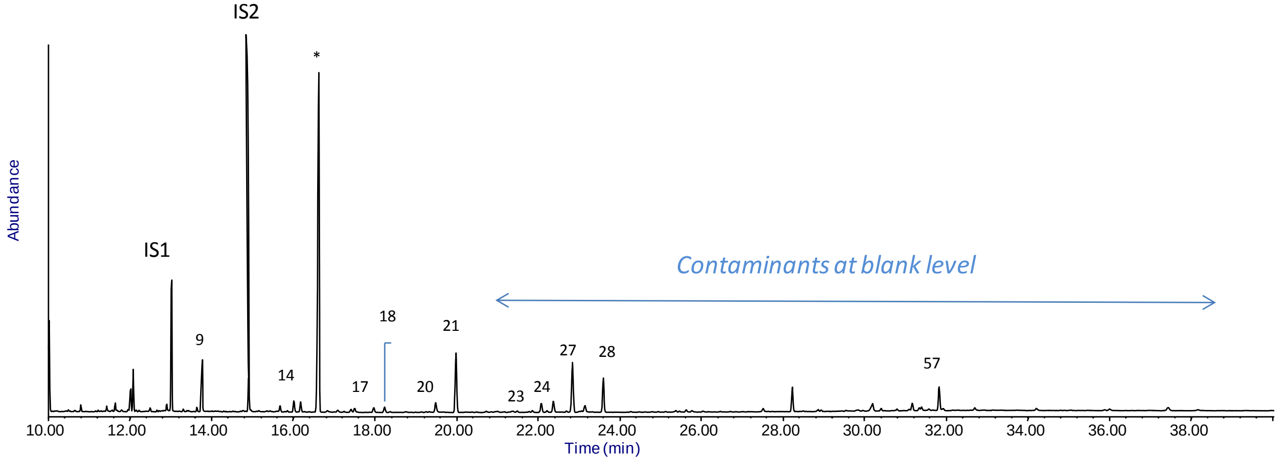

Figure O. Chromatographic profile obtained for lipid-resinous fraction of sample AGO1, M1. The numbers refer to Table 8. IS1 = hexadecane, IS2 = tridecanoic acid, *: phthalate contamination.

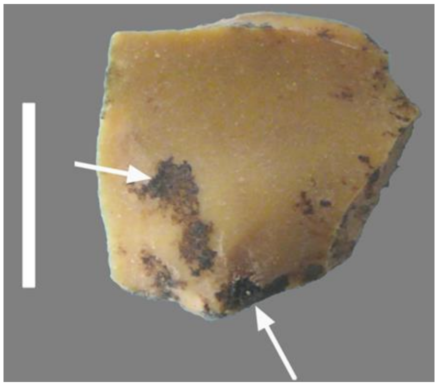

Flake M1 (sample AGO 1) with apparent residue which gave no significant results. The white arrows indicate the analyzed samples. Scale bar = 1cm.

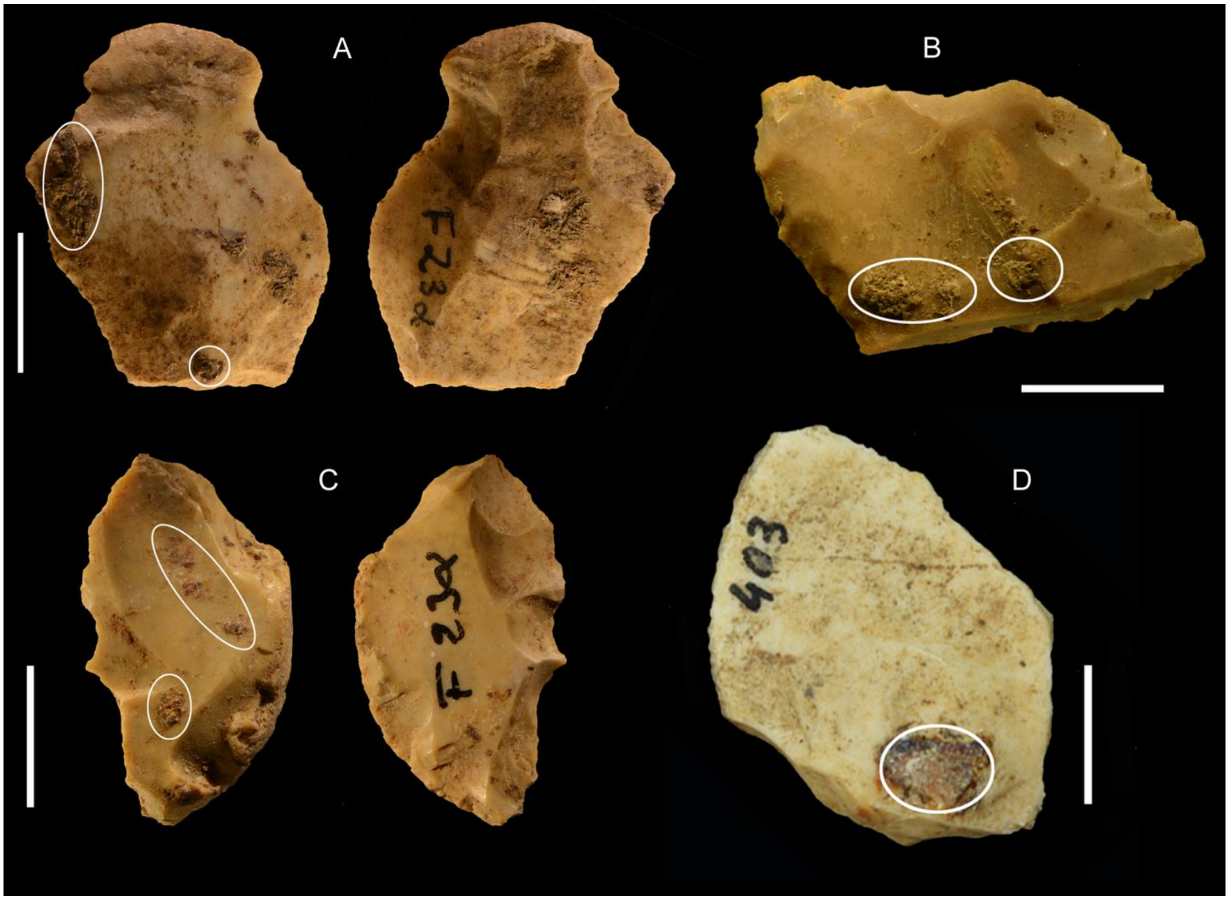

Figure P. Fossellone Cave. Stone artifacts from layer 23 alpha (A-C) and 23 gamma (D) with analyzed residues. The ovals and circles indicate the sampling area. None gave significant results. (A) F2, unretouched flake. (B) F6 broken scraper. (C) F7, denticulate. (D) F10, retouched flake from layer 23 gamma. Scale bar = 1 cm.

References

Andreotti A, Bonaduce I, Colombini MP, Gautier G, Modugno F, Ribechini E. Combined GC/MS Analytical Procedure for the Characterization of Glycerolipid, Waxy, Resinous, and Proteinaceous Materials in a Unique Paint Microsample. Anal Chem. 2006; 78; 4490–4500. internal-pdf://67_andreotti_2006-2810567936/67_Andreotti_2006.pdf.

Garnier N, Cren-Olive C, Rolando C, Regert M. Characterization of Archaeological Beeswax by Electron Ionization and Electrospray Ionization Mass Spectrometry. Anal Chem. 2002; 74: 4868-4877.

Lluveras A, Bonaduce I, Andreotti A, Colombini MP. GC/MS analytical procedure for the characterization of glycerolipids, natural waxes, terpenoid resins, proteinaceous and polysaccharide materials in the same paint microsample avoiding interferences from inorganic media. Anal Chem. 2010; 82: 376–386. doi:10.1021/ac902141m.
